# From Whole-Plant Phytochemistry to Precision Oncology: A Paradigm-Shifting Systematic Review of *Lycium barbarum* L. (Goji Berries)

**DOI:** 10.3390/ph19071115

**Published:** 2026-07-20

**Authors:** Yuanhong Lan, Lanfei Ma, Yuxin Kong, Dina Mahemuti, Congcong Zhang, Yuxiang Zhang, Wenfang Li, Ayitila Maimaitijiang

**Affiliations:** School of Pharmaceutical Sciences, Institute of Materia Medica, College of Life Science and Technology, Xinjiang University, Urumqi 830017, China; 107552505250@stu.xju.edu.cn (Y.L.); 107552403479@stu.xju.edu.cn (L.M.); kongyuxin@stu.xju.edu.cn (Y.K.); dina333@stu.xju.edu.cn (D.M.); 18100993227@163.com (C.Z.); 107552505261@stu.xju.edu.cn (Y.Z.)

**Keywords:** *Lycium barbarum* L., *Lycium barbarum* polysaccharides, medicine–food homology, phytochemical constituents, biological activities, antitumor mechanisms

## Abstract

Medicinal and edible plants are promising resources for low-toxicity therapeutics and precision nutrition. *Lycium barbarum* L. (goji berry), a quintessential medicine–food homologous herb with 2000 years of ethnopharmacological use, has attracted global attention. However, existing studies are limited by fruit-centric bias, lack of correlation between processing methods, component properties and bioactivities, and incomplete antitumor mechanistic understanding. This systematic review establishes a holistic research paradigm that integrates whole-plant resource utilization, processing–property–bioactivity associations, multi-target pharmacology, and clinical translation. We delineate tissue-specific distributions of core bioactives (polysaccharides, phenolics, carotenoids, alkaloids) and their synergistic networks underlying antioxidant, anti-inflammatory, hypoglycemic, and immunomodulatory effects. Critically, we systematically summarize preclinical evidence for the antitumor potential of goji berries, identifying four proposed non-overlapping cell death pathways (apoptosis, cell cycle arrest, ferroptosis, autophagy) and proposed unique roles in reversing multi-drug resistance, alleviating chemoradiotherapy toxicity, and serving as biocompatible nanocarriers, all of which remain predominantly at the preclinical stage. We further propose a three-stage evidence-based roadmap to address key translational bottlenecks. This review bridges the gap between traditional ethnopharmacology and modern precision nutrition, providing a scientific foundation for the sustainable development of the global goji berry industry.

## 1. Introduction

With the advancement of science and technology and the improvement of public health literacy, natural products endowed with both nutritional and medicinal properties have re-emerged as prominent research hotspots [1]. The “medicine–food homology” concept in traditional Chinese medicine, which can be traced back to *Huangdi Neijing Suwen* (Plain Questions of the Yellow Emperor’s Internal Classic) compiled during the period from the Warring States to the Western Han Dynasty, advocates that food acts not only as nourishment but also as therapeutic agents. This notion underscores the dual functions of natural ingredients in maintaining health and preventing and treating diseases [2]. As a typical representative of this concept, *Lycium barbarum* L. (goji berries) is not only a common nourishing food but also a functional food rich in bioactive molecules. Its key components, including *Lycium barbarum* polysaccharides (LBPs), carotenoids, and phenolic compounds, confer unique health-promoting properties, such as immunoregulation [3], delaying cellular senescence [4], regulating glucose [5] and lipid metabolism [6], and protecting retinal function [7].

Despite its promising prospects, objective evaluation is warranted: current clinical translation evidence remains insufficient to support its widespread therapeutic application. Additionally, the in vivo bioavailability of its bioactive substances is hard to precisely control, attributed to the complex gastrointestinal environment, individual differences in gut microbiota metabolism, and the impact of processing methods on component stability.

Notably, the health benefits of goji berries rely on long-term dietary intake and cannot replace standard medical treatment. Nevertheless, their unique multi-target mechanisms—particularly their potential in neuroprotection, immune regulation, and antitumor therapy—render them a crucial bridge between traditional health preservation and modern precision nutrition, underscoring the great potential of natural medicinal plants in the future health industry.

The goji plant is a perennial deciduous shrub belonging to the genus Lycium in the family Solanaceae, which is widely distributed in the arid and semi-arid regions of northwestern China, with Ningxia, Qinghai, Gansu and Xinjiang as the main producing areas. China is the world’s largest producer of goji berries, ranking first in the world in both planting area and output. Ningxia goji berries are particularly famous for their excellent quality and hold an important position in the international market. Although the main producing areas of goji berries are concentrated in northwestern China, their processed products have been exported to North America, Europe, Japan, Southeast Asia and other regions [8].

With the continuous growth of global demand for natural functional foods, goji berries have attracted widespread attention due to their unique nutritional and pharmacological values and have become one of the fastest-growing small berry products in the international market. Moreover, the cultivation of goji berries has gradually expanded to parts of North America, Europe and Central Asian countries, reflecting the continuous improvement of their international recognition and market demand. The bioactive potential of goji berries and their application in the food industry not only have regional characteristics but also conform to the global trends of functional food development and sustainable agriculture and are of great significance for promoting the economic development of arid areas.

The flesh of goji berries is soft and sweet and can be consumed fresh or processed into dried fruits, fruit juices, jams, wine and various nutritional supplements. It is rich in a variety of bioactive components, including LBPs, carotenoids, phenolic compounds, amino acids, vitamins and trace elements. LBPs are regarded as one of their main bioactive substances, exerting multiple pharmacological effects such as regulating immunity, antioxidation, neuroprotection and improving metabolism. In addition to being widely used as food and health products, goji berries have a history of more than 2000 years of application in traditional Chinese medicine and are often used to nourish the liver and kidney, benefit essence and improve eyesight, and enhance physical fitness. Their abundant nutritional components and diverse bioactivities have made goji berries a focus of attention in modern nutrition and pharmacology research, serving as an important carrier connecting traditional wisdom with the modern health industry [9].

Studies have shown that the non-edible parts of the goji plant, including branches, flowers, and the often-overlooked leaves, are also rich in phenolics, flavonoids, polysaccharides, and other secondary metabolites, exhibiting various bioactivities such as antioxidation, anti-inflammation, hypoglycemia, hepatoprotection, and neuroprotection. However, during the large-scale processing of dried goji berries and their juice, a large amount of stems and leaves are usually discarded as waste, resulting in significant resource waste. The resource utilization of goji berry by-products has remarkable economic and ecological value, but there is still a lack of systematic collation on the distribution of bioactive components, health benefits, and potential applications of the whole goji plant. To fill this gap, this review constructs a coherent research context from chemical components to practical applications, linking the main bioactive substances in different parts of the goji plant (fruit, flowers, leaves, stems and root bark) with their potential functions. By integrating scattered research results and combining practical feasibility analysis, this review aims to promote the development of the goji berry industry towards circular economy and clean label directions.

## 2. Search Methodology

This systematic review was conducted in accordance with the Preferred Reporting Items for Systematic Reviews and Meta-Analyses (PRISMA) guidelines.

### 2.1. Literature Search

Comprehensive literature retrieval was performed across five peer-reviewed databases: PubMed, Web of Science, Scopus, ScienceDirect, and China National Knowledge Infrastructure (CNKI). Studies published from database inception to June 2026 were included, with no language restrictions. The core search terms were as follows: (“*Lycium barbarum* L.” OR “goji berry” OR “wolfberry”) AND (“polysaccharides” OR “phytochemical” OR “phenolic” OR “carotenoid” OR “antioxidant” OR “anti-inflammatory” OR “hypoglycemic” OR “immunomodulatory” OR “anticancer” OR “antitumor”). Search syntax was adjusted appropriately for each database to match its retrieval rules.

### 2.2. Inclusion and Exclusion Criteria

Inclusion criteria:

(1) Peer-reviewed original research or review articles focusing on the phytochemistry, biological activities, processing technology, or clinical applications of *Lycium barbarum* L.;
(2) Studies with clear experimental design, quantitative data, and reproducible methods.

Exclusion criteria:

(1) Conference abstracts, letters, editorials, book chapters, and patents without complete experimental data;
(2) Retracted, duplicated, or methodologically flawed studies;
(3) Studies focusing solely on other *Lycium* species (e.g., L. *ruthenicum*) without comparative data for L. *barbarum*.

## 3. Botany

**Figure 1:**
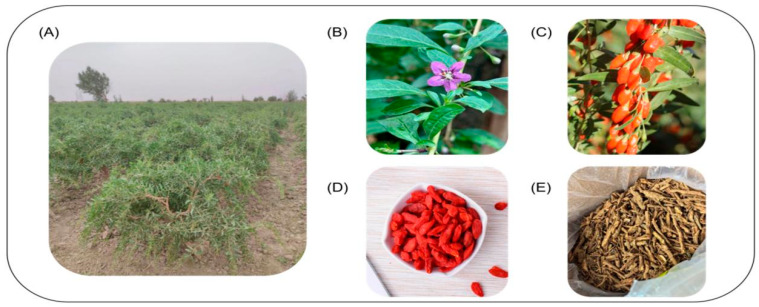
Morphological characteristics of goji berries: (**A**) Plant, (**B**) Flower, (**C**) Fresh fruit, (**D**) Dried fruit, (**E**) Root.

The goji shrub is a multi-branched deciduous shrub with slender and flexible branches, which typically exhibit an arching or pendulous growth habit, bear longitudinal striations on the surface, and are spinose in some cultivars. The leaves are alternate or fascicled, mostly lanceolate or oblong-lanceolate in shape, thick in texture, dark green in color, and possess strong xerophytic adaptability. Flowers are usually borne in leaf axils, with funnel-shaped corollas that are mostly pale purple or light magenta; the stamens are subequal in length to the corolla, and the anthesis generally occurs in summer. The fruit is an ovate or oblong berry, turning bright red or orange-red upon maturity, with a thin and smooth pericarp enclosing soft, succulent flesh and numerous small seeds (Figure 1) [10].

The goji plant bears fruits mainly on current-year spring and summer shoots, which exhibit vigorous growth. As the fruits mature, the branches tend to droop under the increasing fruit load. Similar to grapes, goji berries develop in clusters, which generally require manual cluster-by-cluster or batch harvesting. Among numerous cultivated varieties, goji berries have become the dominant cultivar due to their large fruit size, thick flesh, high sugar content, and outstanding medicinal value. Several elite lines have been bred in Ningxia, Qinghai, Gansu and other regions of China, such as Ningqi 1, Ningqi 5, and Ningqi 14-02, which show excellent performance in yield and quality [11].

Goji berries generally mature from late summer to early autumn, when the dry climate favors sugar accumulation and quality formation. The edible portion is the fleshy berry, with a soluble solids content ranging from 18% to 28%. It is rich in sugars, organic acids, and various bioactive compounds, featuring a sweet and moist taste with a unique flavor, making it highly popular in both domestic and international markets.

## 4. Historical Edible Characteristics

The goji plant has long been regarded as a precious tonic and occupies an important position in traditional medicine. Since ancient times, goji berries have served as a royal tribute in China due to its outstanding health-promoting properties and were highly valued by the imperial court. Li Shizhen, a renowned physician of the Ming Dynasty, highly praised goji berries in his classic work Compendium of Materia Medica, stating that long-term consumption strengthens muscles and bones, lightens the body, and prevents aging, and classified it as a superior-grade medicinal herb [12].

Goji berries are widely used in traditional medicine to nourish the liver and kidney, benefit essence and improve eyesight, nourish blood and calm the mind, and enhance physical strength. In traditional Chinese medicine, they are considered neutral in nature and sweet in taste, with the characteristic of being tonic but not dry, making them suitable for long-term consumption. Historically, goji berries have been commonly used to alleviate blurred vision, soreness and weakness of the waist and knees, dizziness, tinnitus, fatigue, and various discomforts caused by liver–kidney deficiency [13]. Dried goji berries are the most common form for consumption. They can be eaten directly or used for making tea, soup, or porridge or combined with other food ingredients.

In addition to China, goji berries are also used in the traditional medicine of Central Asia, Southeast Asia and other regions and are often used to improve physical strength, protect eyesight and regulate immune function [14,15]. The leaves and root bark (cortex Lycii radicis) of goji also have medicinal value in traditional medicine. Goji leaves can be used to clear heat and improve eyesight, promote fluid production and relieve thirst [16]; cortex Lycii radicis is often used to reduce deficiency-heat, cool blood and lower blood pressure [17].

Overall, multiple parts of the goji plant (fruit, leaves, flowers, stems and root bark) contain abundant bioactive components. In traditional medicine, they are used to maintain visual health, enhance physical strength, regulate immunity, improve sleep and alleviate various chronic discomforts, reflecting the special value of goji berries as a typical medicine–food homologous herb.

## 5. Chemical Constituents

Although the core production areas of goji berries are concentrated in the arid and semi-arid regions of northwestern China, their medicinal and nutritional values have gradually attracted attention from the international scientific community. They enjoy wide popularity not only in their country of origin, China, but also in traditional application regions such as Central Asia and Southeast Asia. Benefiting from their complex and abundant chemical composition, goji berries have become a typical medicine–food homologous fruit with both nutritional and functional properties.

Studies evaluating the nutritional components of goji berries, leaves, and root bark (cortex Lycii radicis) have systematically reported various bioactive substances and nutrients present in this plant. These include the key active constituent LBPs, phenolic compounds (phenolic acids and flavonoids such as chlorogenic acid, caffeic acid, and rutin), saccharides (glucose, fructose, sucrose, etc.), organic acids (citric acid, malic acid, oxalic acid, etc.), fatty acids (unsaturated fatty acids such as linoleic acid and linolenic acid), terpenoids (carotenoids), various essential amino acids (leucine, lysine, phenylalanine, etc.), macroelements (K, Ca, Mg, Na, P), vitamins (vitamin C, vitamin E, carotenoids, etc.), and trace elements (Fe, Zn, Cu, Se, etc.) [18]. Table 1 summarizes studies on analytical methods related to chemical constituents in the fruit, leaves, and root bark of the goji plant. The selected compounds represent the main nutritional and functional components in different parts of the goji plant.

In goji berries, carbohydrates are the dominant components. The soluble solids content accounts for 18–28% of the fresh weight, with fructose and glucose serving as the main sweetening agents. As a characteristic bioactive constituent, LBPs constitute 5–12% of the fruit dry weight. They are not only an important nutritional substance but have also been verified to possess significant activities including antioxidation, immunoregulation, hypoglycemic effect, neuroprotection, and improvement of intestinal flora balance [19]. Organic acids in the fruit (e.g., citric acid) contribute to flavor and taste, while carotenoids (e.g., zeaxanthin and lutein) are closely associated with vision protection. The abundant amino acids, vitamins, and trace elements provide essential nutrients for the human body and help maintain normal physiological functions. In comparison, the leaves and root bark of goji berries also exhibit high utilization value. The leaves are rich in phenolic compounds and dietary fiber, showing favorable antioxidant and anti-inflammatory activities. The root bark (cortex Lycii radicis) contains unique constituents such as betaine and lyciumamide, as well as various phenolic compounds, exerting pharmacological effects including reducing deficiency-heat, cooling blood, lowering blood pressure, and antioxidation.

These bioactive compounds, widely distributed in various parts of the goji plant, not only form the core basis of its nutritional and health-promoting values but also provide important support for the development of natural extracts, research and development of functional foods, and sustainable utilization of goji berry by-products, demonstrating significant academic research value and industrial application prospects.

**Table 1:** Summary of detected phytochemicals, extraction strategies, and analytical approaches in the fruit, leaves, flowers, stems and root bark of goji plant.

Compound Category	Plant Part	Detected Components	Extraction Solvent and Method	Analytical Technique	Reference
Sugars and polysaccharides	Fruit	Fructose, glucose, sucrose, LBPs	Water; ultrasound-assisted extraction; hot-water extraction + ethanol precipitation	HPLC-RI; phenol–sulfuric acid method; ATR-FT-IR	[20]
	Stems	Fructose; glucose; sucrose	Aqueous extraction system	HPLC-RI	[20]
	Leaves	HG, RG-I pectins; fucose, rhamnose, arabinose, galactose, glucose, xylose	CDTA/Na_2_CO_3_/NaOH sequential extraction; ethanol precipitation; anion-exchange chromatography	HPAEC-PAD, SEC-MALLS-RI, FT-IR, NMR, AFM	[21]
	Flowers	LBPs; glucose, galactose, arabinose	Hot-water extraction (80 °C); ultrasonic-assisted extraction; 80% ethanol precipitation; Sevag deproteinization	UV–Vis; HPLC-ELSD; GC-MS	[22]
	Root bark	LBPs; scopolin, linarin, phenethyl alcohol glycosides	Hot-water reflux; macroporous resin fractionation; solvent extraction	HPLC-DAD; LC-MS/MS; HPLC-ELSD; GC-MS	[23]
Phenolics	Fruit	Caffeic acid; chlorogenic acid; quercetin; P-coumaric acid; 5-O-caffeoylquinic acid; caftaric acid; ferulic acid; catechin; rutin; isoquercitrin; hyperoside; quercitrin; kaempferol	Methanol–water (80:20, *v*/*v*); shaking extraction; ultrasound-assisted extraction	LC-DAD-ESI/MS^n^; Folin–Ciocalteu assay; ATR-FT-IR	[24]
	Leaves	Quercetin; rutin; chlorogenic acid	90% methanol; shaking extraction	Folin–Ciocalteu assay; UPLC–MS/MS	[25]
	Stems	5-O-caffeoylquinic acid; galloylquinic acid; caffeic acid; sinapic acid; kaempferol-3-O-rutinoside; rutin	Methanol–water mixture	LC-DAD-ESI/MS^n^	[20]
	Flowers	Epicatechin; gentisic acid; ferulic acid; rutin; chlorogenic acid; gallic acid; caffeic acid; quercetin; kaempferol	Free phenolics: acetone/methanol–water mixture, homogenization-assisted solvent extraction; bound phenolics: NaOH hydrolysis + ethyl acetate extraction	HPLC-DAD-MS; LC-MS/MS; UV–Vis spectrophotometry	[22]
	Root bark	Epicatechin; gentisic acid; 4-methylcatechol; ferulic acid; rutin; caffeic acid; sinapic acid; dihydrocaffeic acid; vanillic acid; isovanillic acid; P-hydroxybenzoic acid; kaempferide; apigenin; luteolin; 7-hydroxycoumarin; scoparone; scopoletin; dihydro-N-caffeoyltyramine; chlorogenic acid ethyl ester	Free phenolics: 70–95% ethanol, ethyl acetate, reflux/ultrasonic-assisted extraction; bound phenolics: alkali hydrolysis + solvent extraction	HPLC-DAD-MS; LC-MS/MS; UV–Vis spectrophotometry	[23]
Organic acids	Fruit	Oxalic acid, citric acid, succinic acid, formic acid, acetic acid, hexanoic acid, isovaleric acid, pentanoic acid	Aqueous extraction; headspace SPME; HS-GC-IMS incubation	UFLC-PDA; GC × GC-TOFMS; HS-GC-IMS	[26,27,28]
	Stems	Oxalic acid, quinic acid, malic acid	Aqueous extraction system	UFLC-PDA	[20]
	Root bark	Cinnamic acid, indole-3-carboxylic acid, azelaic acid, (2E,4S)-4-hydroxy-2-nonenoic acid, taurine	95% ethanol, ethyl acetate; solvent extraction; reflux extraction	HPLC-DAD-MS; GC-MS	[23]
Fatty acids	Fruit	Linoleic acid, palmitic acid, oleic acid, stearic acid, α-linolenic acid, arachidic acid, behenic acid, lignoceric acid, palmitoleic acid, gondoic acid	Methanol/hexane, methyl esterification; petroleum ether, Soxhlet extraction + trans-esterification	GC-MS (FAME); GC-FID	[24]
	Stems	Saturated fatty acids (SFAs, mainly palmitic acid and lignoceric acid), monounsaturated fatty acids (MUFAs), polyunsaturated fatty acids (PUFAs, mainly linolenic acid), in total 18 fatty acids	Petroleum ether; Soxhlet extraction + trans-esterification	GC-FID	[20]
	Root bark	Linoleic acid, linolenic acid, melissic acid, palmitic acid, stearic acid, oleic acid, (10E,12Z,9S)-9-hydroxyoctadecadienoic acid, (10E,12Z,15Z,9S)-9-hydroxyoctadecatrienoic acid, octadecyl ferulic acid	Petroleum ether, n-hexane; Soxhlet extraction; solvent extraction	GC-MS; LC-MS/MS; HPLC-DAD-MS	[23]
Amino acids and nucleosides	Flowers	Total free amino acids (high essential amino acid ratio); uridine, guanosine, adenosine (significantly higher than leaves)	0.1 mol/L hydrochloric acid; ultrasonic-assisted extraction; homogenate extraction	HPLC-DAD; UPLC-MS/MS; amino acid analyzer	[22]
	Leaves	Free amino acids (high umami amino acid ratio); stable nucleoside content (lower than flowers)	Methanol–water mixture; homogenate extraction	UPLC-MS/MS	[22]
	Root bark	L-phenylalanine; lyciumin A–D; novel cyclic octapeptide	Deionized water; hot-water reflux extraction; macroporous resin fractionation	HPLC-Q-TOF-MS; LC-MS/MS; amino acid analyzer	[23]
Terpenoids and carotenoids	Fruit	D-limonene, α-pinene, β-ocimene, 1,8-cineole; zeaxanthin, lutein (esterified forms)	Headspace SPME; 80% acetone extraction	GC × GC-TOFMS; HS-GC-IMS; spectrophotometry	[26]
	Leaves	Total carotenoids; chlorophylls a, b	80% acetone extraction	Spectrophotometry	[25]
	Root bark	Ursolic acid, β-sitosterol, stigmasterol, lanosterol, cycloartenol, campesterol	Petroleum ether, 95% ethanol; Soxhlet extraction; reflux extraction	GC-MS; LC-MS/MS; HPLC-ELSD	[23]
Vitamins	Fruit	Vitamin C, zeaxanthin dipalmitate, total carotenoids	Water; ultrasound-assisted extraction	ATR-FTIR; spectrophotometry	[24]
	Leaves	Chlorophylls (a, b), carotenoids	80% acetone extraction	Spectrophotometry	
	Root bark	Nicotinic acid (vitamin B_3_), choline (vitamin B_4_), zeaxanthin dipalmitate, melatonin; kukoamine A/B, betaine, lyciumamide	Methanol–water mixture; ultrasonic-assisted solvent extraction; water decoction	HPLC-UV; LC-MS/MS; GC-MS; spectrophotometry	[25]
Others	Fruit	Aldehydes (hexanal, (E)-2-hexenal, nonanal, furfural, β-cyclocitral, safranal), alcohols (1-hexanol, 4-hexen-1-ol, 2-hexen-1-ol, linalool, phenethyl alcohol), ketones (geranylacetone, 2-undecanone, 6-methyl-5-hepten-2-one, β-ionone, acetoin), esters (ethyl acetate, hexyl acetate, methyl salicylate, ethyl pentanoate), furans (2-pentylfuran, 2-ethylfuran)	SPME (50/30 μm DVB/CAR/PDMS); 60 °C equilibration 20 min, 50 °C adsorption 35 min, 250 °C desorption 3 min	GC × GC-TOFMS, GC–MS-O, HS-GC-IMS	[26]
	Leaves	Short-chain fatty acids (acetate, propionate, butyrate, isobutyrate, valerate, isovalerate); probiotics (Bacteroides, Bifidobacteria, Lactobacilli)	In vitro human fecal fermentation	GC-FID	[27]
	Root bark	Alkaloids: kukoamine A, kukoamine B, lycinmamide, betaine, atropine, scopolamine, calystegines, allantoin, adenine; Anthraquinones: emodin, physcion, 2-methyl-1,3,6-trihydroxy-9,10-anthraquinone; vanillin, isovanillin, periplogenin	Methanol–water mixture, 70–95% ethanol, deionized water, chloroform, ethyl acetate; ultrasonic-assisted solvent extraction (30 min, 60 °C), reflux extraction, water decoction, solvent extraction, homogenate extraction	HPLC-UV, LC-MS/MS, HPLC-Q-TOF-MS, UPLC-MS/MS	[23]

HPLC-RI—high-performance liquid chromatography coupled with refraction index detector; ATR-FT-IR—attenuated total reflection Fourier transform infrared spectroscopy; HPAEC-PAD—high-performance anion-exchange chromatography with pulsed amperometric detection; SEC-MALLS-RI—size-exclusion chromatography coupled with multi-angle laser light scattering and refractive index detection; FT-IR—Fourier transform infrared spectroscopy; NMR—nuclear magnetic resonance spectroscopy; AFM—atomic force microscopy; HPLC–UV–Vis—high-pressure liquid chromatography with UV/visible detector; HPLC-ELSD—high-performance liquid chromatography with evaporative light scattering detection; SPME–GC–MS—gas chromatography–mass spectrometry combined with solid-phase microextraction; HPLC–DAD—high-performance liquid chromatography with diode-array detection; LC-MS/MS—liquid chromatography–tandem mass spectrometry; GC–MS—gas chromatography with mass detector; UPLC–MS/MS—ultra-performance liquid chromatography–tandem mass spectrometry; LC-DAD-ESI/MS—liquid chromatography–diode-array detection–electrospray ionization–multi-stage mass spectrometry; HPLC–DAD–MS—high-performance liquid chromatography with diode-array detection and mass spectrometry; GC × GC-TOFMS—comprehensive two-dimensional gas chromatography–time-of-flight mass spectrometry; GC–MS-O—gas chromatography–mass spectrometry–olfactometry; HS-GC-IMS—headspace gas chromatography–ion mobility spectrometry; HPLC–PDA—high-performance liquid chromatography with photodiode-array detection; GC–FID—gas chromatography with flame ionization; UHPLC—ultra-high-performance liquid chromatography; GC–MS—gas chromatography with mass detector; HPLC–FD—high-performance liquid chromatography with fluorescence detector; GC—gas chromatography; HPLC-Q-TOF-MS—high-performance liquid chromatography–quadrupole time-of-flight mass spectrometry.

### 5.1. Lycium barbarum Polysaccharides

**Figure 2:**
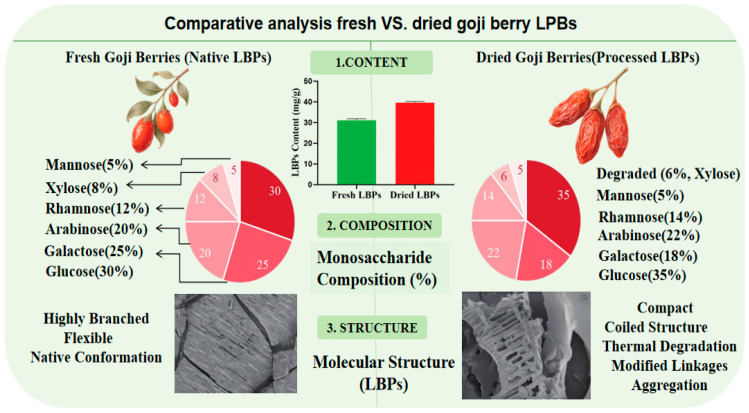
Conceptual schematic of structural and compositional differences between fresh and dried goji berry LBPs, synthesized based on published experimental data from multiple studies [30,31,32]. This schematic is a conceptual summary synthesized from published experimental data and does not present original experimental results of this work.

LBPs are the most representative active components of goji berries. They are water-soluble polysaccharides beneficial to human health and serve as the core material basis for the tonic and health-promoting effects of goji berries. As secondary metabolites of plants, LBPs are a class of structurally complex heteropolysaccharides, mainly composed of various monosaccharides, including arabinose, glucose, galactose, rhamnose, and xylose linked by glycosidic bonds. Some are also conjugated with proteins to form glycoprotein complexes, exhibiting a variety of unique biological functions [29]. Studies have shown that significant differences exist in the content, composition, and structure of LBPs between fresh and dried goji berries (Figure 2). In fresh goji berries, LBPs mainly exist as water-soluble crude polysaccharides, dominated by heteropolysaccharides with arabinose–galactose backbones, together with a small amount of acidic polysaccharides modified by glucuronic acid [30].

Pretreatment methods and drying processes significantly affect the extraction efficiency, compositional stability, and biological activity of LBPs in goji berries. Enzymatic pretreatment (e.g., combined treatment with cellulase and pectinase) can effectively disrupt the cell wall structure of goji berries, promote the release of polysaccharides, and markedly increase the extraction yield of total polysaccharides. Meanwhile, it can induce moderate degradation of some macromolecular polysaccharides, increase the proportion of low-molecular-weight polysaccharide fractions, and thereby improve their bioavailability. Compared with fresh goji berries, dried goji berries exhibit distinctive compositional characteristics of LBPs. Different drying methods such as hot-air drying and vacuum freeze drying lead to changes in the molecular weight distribution of polysaccharides. Among them, vacuum freeze drying best preserves the original structure and active components of polysaccharides, whereas high-temperature hot-air drying may cause partial degradation or polymerization of polysaccharides, altering the molar ratios of constituent monosaccharides such as arabinose and galactose [32]. In addition, LBPs in dried goji berries form a more stable spatial conformation, with slightly reduced water solubility but enhanced tolerance to acidic and alkaline environments. This property facilitates the subsequent processing and application of LBPs. These findings reflect the differences in composition and structure of LBPs between fresh and dried goji berries, indicating that processing methods exert significant effects on the stability, molecular weight distribution, and bioavailability of LBPs, which may further regulate their functional properties in health-related applications such as immunoregulation, antioxidant activity, and hypoglycemic effects [33].

Obvious differences also exist in the type, content, and structure of LBPs in different tissues of the goji plant. For example, LBPs in goji berries are mainly neutral heteropolysaccharides with a high degree of sugar chain branching and low bound protein content (usually below 5%); polysaccharides in goji leaves are dominated by acidic polysaccharides containing abundant glucuronic acid residues, and their rhamnose content is significantly higher than that in fruit polysaccharides; polysaccharide fractions in goji root bark are more complex. Besides arabinose–galactose-type heteropolysaccharides, they also contain a small amount of xylan-type polysaccharides, and the total polysaccharide content is remarkably lower than that in fruits [34]. The total polysaccharide content differs significantly among different tissues of the goji plant. Numerous studies have demonstrated that the total polysaccharide content is the highest in goji berries, reaching 8–12% of the dry weight in high-quality goji berries produced in Ningxia. Next are goji leaves, with a total polysaccharide content of approximately 3–6% of dry weight. Goji root bark has the lowest total polysaccharide content, accounting for only 1–2% of dry weight. Meanwhile, variations in fruit polysaccharide content exist among different goji berry varieties. For instance, elite cultivars such as “Ningqi 1” and “Ningqi 5” show significantly higher fruit polysaccharide contents than ordinary varieties. These results suggest that the differences in LBP content among different parts and varieties of goji berries may lead to distinct intensities of biological activity, especially in immunoregulation and neuroprotection. This indicates that goji berries remain the optimal part for the extraction and utilization of LBPs, while goji leaves, as processing by-products, also possess certain development potential [12,35,36].

The selection of extraction solvent is one of the key factors affecting the content and purity of total polysaccharides in goji berries. When extracting polysaccharide compounds from goji berries using different solvents, the extraction yield decreases in the following order: water extract, dilute ethanol solution (20–40%) extract, concentrated ethanol solution (above 60%) extract, and methanol extract, which is consistent with the water-soluble property of LBPs. Similarly, studies on goji leaf polysaccharides have shown that hot-water extraction (80–90 °C) is the most effective solvent-based method, yielding polysaccharides with high purity and low contents of impurities such as mixed proteins and pigments, followed by dilute sodium hydroxide solution. In contrast, acidic solution extraction may cause cleavage of polysaccharide glycosidic bonds, reducing the extraction yield and biological activity [37]. For goji berries, water and dilute ethanol solutions consistently achieve the highest total polysaccharide contents. Moreover, extraction with dilute ethanol solutions can effectively reduce the coextraction of impurities such as proteins, simplifying the subsequent purification process [38].

These findings are highly consistent with the chemical properties of LBPs. As water-soluble heteropolysaccharides, LBPs display higher solubility in polar solvents such as water and dilute ethanol but very low solubility in less polar solvents, including methanol and ethyl acetate. Although the use of solvents such as concentrated ethanol and methanol has been occasionally reported, it usually results in low extraction efficiency and carries solvent residue risks, making it less relevant for food, nutraceutical, or clinical applications. Therefore, future studies are encouraged to prioritize food-grade, environmentally friendly, and physiologically relevant extraction methods such as water extraction and dilute ethanol extraction to improve the extraction efficiency and application safety of LBPs. In addition, extraction technology also significantly influences the total polysaccharide content in goji berries. For example, when extracting polysaccharide compounds from goji berries using different techniques, the total polysaccharide content follows the order: ultrasound-assisted extraction (power 200–300 W, extraction time 30–60 min) > microwave-assisted extraction (power 500–600 W, extraction time 5–10 min) > high-pressure extraction (300–500 MPa, extraction time 10–20 min) > conventional hot-water extraction (magnetic stirring, extraction time 2–3 h) [39].

Furthermore, studies have confirmed that, under the same solvent and time conditions, ultrasound-assisted extraction yields a higher polysaccharide content than traditional hot-water extraction, and the extracted polysaccharides exhibit superior biological activities (e.g., immunoregulatory activity), indicating better extraction efficiency and quality. Notably, the polysaccharide content obtained by conventional hot-water extraction for 2 h is only equivalent to that by ultrasound-assisted extraction for 30 min. Moreover, ultrasound-extracted polysaccharides show higher purity, with a reduction in mixed protein content of approximately 30–40%. These findings underscore the importance of extraction methods in interpreting data on LBP composition and activity, while providing technical references for the efficient industrial extraction of LBPs [40].

At present, the main techniques for extracting polysaccharides from goji berries include hot-water extraction, enzymatic extraction, ultrasound-assisted extraction, microwave-assisted extraction, and combined extraction methods integrating multiple technologies. Each extraction method has its own advantages and disadvantages, as shown in Table 2.

### 5.2. Phenolic Compounds

**Figure 3:**
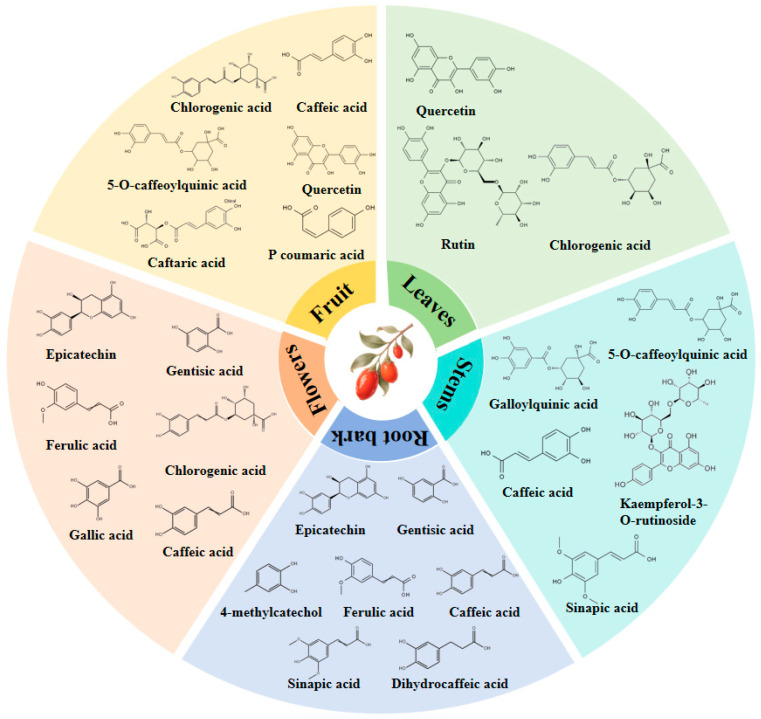
Representative phenolic compounds identified in different parts of goji plant and their chemical structures. All indicates that the compound is present in all tested tissues, including fruit, leaves, flowers, stems and root bark.

Phenolic compounds represent another pivotal class of bioactive constituents in Lycium species. As non-nutritive secondary plant metabolites, they function synergistically with LBPs to underpin the nutritional and therapeutic value of goji berries. Characterized structurally by aromatic rings bearing one or more hydroxyl groups, these compounds are diverse, primarily classified into phenolic acids, flavonoids, and tannins. The major representative bioactive components across distinct plant tissues are summarized in Figure 3. They exhibit a broad spectrum of biological activities, including antioxidant, anti-inflammatory, neuroprotective, and cardioprotective properties [41].

The retention and transformation of these phenolic moieties are profoundly influenced by pretreatment and drying methodologies. Enzymatic pretreatment—particularly the combination of polyphenol oxidase inhibitors with cellulase—effectively mitigates oxidative degradation while disrupting cell wall integrity to facilitate phenolic release. This approach significantly enhances the extraction yield of total phenolics, with marked increases observed for chlorogenic acid and rutin, whereas ferulic acid remains relatively unaffected [42,43]. Regarding drying processes, vacuum freeze drying preserves the native phenolic profile to the greatest extent, although slight hydrolysis of flavonoid glycosides may occur. Conversely, the drying process can subtly alter the polarity of phenolic compounds, thereby influencing the selection of subsequent extraction solvents [44].

Significant variations in phenolic composition and concentration exist across different anatomical parts of the Lycium plant. Notably, leaves exhibit the most diverse phenolic profile, being rich in chlorogenic acid, isochlorogenic acid, luteolin, and apigenin. Their total phenolic content reaches 5–8% of dry weight, significantly higher than that of the fruit. In contrast, the fruit primarily contains rutin, chlorogenic acid, and caffeic acid, with total phenolics ranging from 1–3% of dry weight, exhibiting varietal dependence; superior cultivars such as “Ningqi 1” and “Ningqi 5” from Ningxia demonstrate significantly higher phenolic content than standard varieties. The root bark is characterized by coumarin derivatives and minor phenolic acids, with total phenolics constituting approximately 2–4% of dry weight [45].

Comparative analyses consistently rank the total phenolic content as follows: leaves > root bark > fruit. This hierarchical distribution correlates directly with the potency of biological activities. Phenolic extracts derived from leaves demonstrate markedly superior antioxidant capacity—measured by DPPH radical scavenging and total reducing power—compared to extracts from fruit and root bark. This suggests that goji leaves, often regarded as by-products, possess considerable potential for development as natural antioxidants. Solvent selection is a critical determinant of extraction efficiency and purity. Due to their polyhydroxy structure and high polarity, phenolics exhibit a strong correlation between solubility and solvent polarity. While methanol (50–70%) generally yields the highest extraction efficiency in laboratory settings, 70% ethanol is preferred for industrial food applications due to its optimal balance of extraction efficacy and safety, yielding high-purity extracts with minimal interference from sugars or proteins. Similarly, although 70% methanol maximizes yield from leaves, 70% ethanol is favored for industrial scalability and safety compliance [46].

Moreover, the extraction methodology significantly influences efficiency. A comparative hierarchy of techniques for fruit-derived phenolics ranks as follows: UAE (250–350 W, 20–40 min) > MAE (400–600 W, 3–8 min) > high-pressure extraction (400–600 MPa, 15–25 min) > conventional solvent extraction (1–2 h). Under identical solvent and time conditions, UAE enhances total phenolic yield by 30–50% compared to conventional methods. This enhancement is attributed to the cavitation effect, which disrupts cell walls, promotes mass transfer, and reduces oxidative degradation. Crucially, 30 min of ultrasound-assisted extraction achieves equivalent yields to 1.5 h of conventional stirring, while simultaneously reducing pigment impurities. These insights underscore the paramount importance of extraction methodology in Lycium phenolic research, providing a robust technical foundation for efficient and green extraction protocols [47].

### 5.3. Carbohydrates

In Lycium plants, the distribution of carbohydrate components varies significantly across different tissues. The total sugar content in the fruit pulp is considerably higher than in seeds and peels; accordingly, most studies have focused on the composition and properties of carbohydrates in the pulp, whereas investigations on sugars in seeds and peels remain relatively limited. Research data indicate that the glucose content in dried goji berry pulp is approximately 3.2-fold higher than in seeds, fructose about 3.5-fold higher, and sucrose nearly 186-fold higher. Such pronounced differences in sugar levels directly determine the utilization strategies of distinct goji berry tissues: pulp, with its high sugar content, serves as the primary material for consumption and processing, whereas seeds and peels, due to their low sugar proportions, are mostly regarded as by-products for exploring other bioactive constituents [35].

Notably, different processing treatments of goji berry pulp markedly alter the composition and ratio of its carbohydrate components, thereby influencing the flavor profile and nutritional value of the final products. For instance, studies have revealed that distinct concentration techniques during goji berry juice processing, including vacuum concentration and atmospheric concentration, exert differential effects on sucrose content. High-temperature atmospheric concentration induces slight degradation of sucrose, leading to an increased proportion of reducing sugars (glucose and fructose) and a reduction in certain functional oligosaccharides, indirectly diminishing the nutritional value of the juice. Dynamic changes in carbohydrate composition are also observed during the drying of fresh goji berries. On the one hand, water loss during dehydration results in a relative increase in monosaccharide concentrations. On the other hand, partial polysaccharides may undergo depolymerization and repolymerization, while the smooth regions of pectin structures in fresh fruit are disrupted by dehydration, further affecting the existing forms and extractable properties of carbohydrates [48,49,50].

Different extraction methods also alter the relative abundance of carbohydrate components in goji berry pulp, primarily due to variations in the degree of cell disruption and action mechanisms, which in turn affect the release efficiency of different sugar types. For example, compared with conventional solvent extraction, the application of ultra-high-pressure-assisted enzymatic extraction leads to a significantly higher relative percentage of rhamnose in the extracts, accompanied by reduced proportions of mannose and glucose. This phenomenon may be attributed to the selective cleavage of specific glycosidic linkages by enzymatic hydrolysis and the disruption of intracellular carbohydrate complexes under ultra-high pressure.

Furthermore, the content and composition of carbohydrates undergo regular dynamic changes during the development of goji berry pulp. In the immature green-fruit stage, sucrose-metabolizing enzyme activity is relatively high in goji berry pulp, and large amounts of sucrose are hydrolyzed into glucose and fructose to maintain intracellular osmotic balance and meet physiological demands during early fruit development. At this stage, glucose is the most abundant sugar, whereas sucrose content remains at the lowest level. As the fruit gradually ripens and enters the red-fruit stage, sucrose activity gradually decreases, whereas sucrose synthase activity is enhanced, resulting in a sharp increase in sucrose accumulation, which ultimately becomes a dominant sugar component in mature goji berry pulp. Such dynamic shifts in carbohydrates during fruit development provide an important theoretical basis for precisely regulating goji berry quality and determining the optimal harvest time [51].

### 5.4. Organic Acids

Organic acids are widely distributed in all fruits, and their composition and content vary significantly under the regulation of fruit species, cultivar, growing environment and developmental stages. Some fruits are rich in malic acid, citric acid, quinic acid and other organic acids, while phenolic acids and ascorbic acid are commonly present in various fruits. Organic acids regulate fruit ripening and quality formation through multiple pathways, and their types and concentrations significantly affect the sour–sweet taste, color and aroma synthesis of fruits [52].

Studies have shown that the main organic acids in goji berries include citric acid, malic acid, oxalic acid, succinic acid, quinic acid and ascorbic acid, among which citric acid and malic acid are the most abundant and form the core of the characteristic sour–sweet flavor of goji berries [53]. In terms of tissue distribution, the contents of citric acid, malic acid and ascorbic acid in goji berry pulp are significantly higher than those in seeds and peels. As an important water-soluble antioxidant, ascorbic acid contributes greatly to the health-promoting value of goji berries. Quinic acid, one of the characteristic organic acids in goji berries, acts as a precursor for the biosynthesis of some phenolic antioxidants, but its low content results in a limited direct contribution to the overall antioxidant activity.

Processing treatments can significantly alter the composition and content of organic acids in goji berry pulp, among which fermentation exerts the most remarkable effect. Research data indicate that the total organic acid content in unfermented goji berry pulp is approximately 18.35 mg/g DW, which is significantly increased to 52.68 mg/g DW after fermentation. Specifically, the contents of lactic acid, acetic acid and succinic acid increase substantially, whereas those of oxalic acid and malic acid decrease obviously. Such changes not only influence the flavor of fermented goji plant products but also may enhance their antibacterial activity [54].

In addition, cultivation practices also regulate the organic acid content of goji berries. Studies have found that bagging treatment after flowering significantly reduces malic acid content in the pulp, which may be attributed to the altered microenvironment (e.g., light and temperature), accelerated fruit ripening and enhanced degradation metabolism of organic acids caused by bagging. This finding provides practical references for optimizing the flavor quality of goji berries through cultivation regulation [55].

### 5.5. Fatty Acids

Based on the presence or absence of carbon–carbon double bonds in their molecular structure, fatty acids can be divided into two major categories: saturated fatty acids and unsaturated fatty acids. Among them, ω-6 polyunsaturated fatty acids (ω-6 PUFAs) and ω-3 polyunsaturated fatty acids (ω-3 PUFAs) are essential fatty acids that cannot be synthesized by the human body and must be obtained from the diet. These fatty acids play key roles in regulating human metabolism and maintaining cardiovascular health and are therefore widely regarded as important functional components for functional foods and nutritional supplements.

Studies have shown that goji berries and various tissues of the plant are rich in fatty acids, with unsaturated fatty acids as the dominant components, which constitute an important material basis for the fat-soluble nutritional and health-promoting effects of goji berries. In terms of tissue distribution, goji berry seeds are the main accumulation site of fatty acids, with a significantly higher fatty acid content than pulp and peel. In addition, squalene, a characteristic fat-soluble component in goji berry pulp, exhibits physiological functions, including blood lipid regulation, antioxidant activity, and immune enhancement, and exerts positive effects in the prevention of cardiovascular diseases [56].

The main fatty acid components in goji berry seeds and peel are unsaturated fatty acids. These fatty acids possess important physiological activities such as inhibiting platelet aggregation, reducing blood cholesterol and triglyceride levels, and suppressing lipid peroxidation, which are of great significance for maintaining cardiovascular health. Existing studies have confirmed that goji berry seeds contain abundant unsaturated fatty acids, especially oleic acid, linoleic acid, and α-linolenic acid, which together account for more than 78.6% of the total fatty acids. The seeds are also rich in ω-3 and ω-6 PUFAs with a balanced ratio, meeting human nutritional requirements [57].

The content and composition of unsaturated fatty acids in goji berry seeds are also closely related to cultivar characteristics and postharvest regulation measures, thereby affecting the storage quality and nutritional value of goji berries. Studies have found that seeds of the authentic Ningxia cultivar “Ningqi 1” maintain a higher degree of fatty acid unsaturation compared with common varieties. This characteristic helps maintain the structural integrity of the seed cell membrane system, reduces lipid oxidation during storage, and thus delays quality deterioration of goji berries. In addition, exogenous regulation measures can also affect the fatty acid composition of goji berries. For example, preharvest spraying of appropriate vitamin E significantly increases the content of unsaturated fatty acids in goji berry seeds, especially the proportion of α-linolenic acid, thereby improving the storability and nutritional quality of goji berries [58,59].

### 5.6. Amino Acids

Amino acids represent important nutritional components in goji berries, and their composition and content are directly related to the nutritional value and flavor characteristics of goji berries. Studies have shown that fresh goji berry pulp contains eight kinds of essential amino acids for humans and more than 10 kinds of non-essential amino acids, among which free amino acids are abundant, especially glutamic acid, aspartic acid and alanine. These three amino acids account for more than 40% of the total free amino acids, constituting the key material basis for the umami and sweet taste of goji berries [60].

Drying is one of the most common processing methods for goji berries, which significantly changes the composition and content of amino acids in goji berry pulp. Research has found that the aqueous extract of dried goji berry pulp contains nearly 20 amino acids, with glutamic acid still ranking first, followed by alanine, arginine and leucine. During the drying process, high temperature induces the degradation of free amino acids and the Maillard reaction, resulting in a significant decrease in the total content of free amino acids in dried goji berries compared with fresh pulp. Meanwhile, the relative contents of sweet amino acids (such as alanine and glycine) increase remarkably, which is one of the important reasons why dried goji berries exhibits stronger sweetness and aromatic flavor than fresh goji berries [61].

In addition to drying, fermentation also exerts a significant influence on the amino acid composition in goji berry pulp. After fermentation, the total content of free amino acids in dried goji berry pulp shows a downward trend, which may be related to the metabolic activities of microorganisms during fermentation. Meanwhile, complex reactions such as Maillard reaction and enzymatic transformation also consume part of the free amino acids. In terms of specific components, the contents of bitter-related amino acids (such as phenylalanine and tyrosine) decrease significantly after fermentation, which effectively improves the taste of fermented goji berry products. In contrast, the contents of some amino acids associated with antioxidant activity (such as aspartic acid and cysteine) increase, further enhancing the nutritional and health-promoting value of fermented goji berry products [62].

Furthermore, goji berry cultivar and geographical environment also affect the composition and content of amino acids. The total contents of essential amino acids in fresh pulp of authentic Ningxia cultivars “Ningqi 1” and “Ningqi 5” are significantly higher than those in common varieties, especially for key essential amino acids such as leucine and lysine. Environments with sufficient sunlight and large diurnal temperature differences contribute to the accumulation of amino acids in goji berries, which is one of the important reasons for the outstanding nutritional value of goji berries produced in Ningxia. These findings provide an important theoretical basis for goji berry breeding, processing optimization and the construction of high-quality production areas [60].

### 5.7. Terpenoids

Terpenoids are important fat-soluble bioactive components in goji berries, widely distributed in fruits, leaves and peels, and serve as one of the key material bases for the flavor and health-promoting value of goji berries. According to the number of isoprene units, they can be classified into monoterpenes, triterpenes and carotenoids (tetraterpenes), with diverse biological functions.

Carotenoids are the most abundant terpenoids in goji berries and the main source of the red color of fruits, mainly including zeaxanthin and lutein, which account for more than 60% of the total carotenoids and mostly exist in stable esterified forms. These components possess excellent antioxidant activity, protect the retina, regulate immunity, and exert beneficial effects on cardiovascular health [63].

Goji berries also contain triterpenoids such as oleanolic acid and ursolic acid, which are mainly concentrated in the peel and leaves and exhibit anti-inflammatory, hepatoprotective and hypoglycemic activities. In addition, monoterpenes such as linalool are present in low contents but are crucial for the formation of the unique aroma of goji berries [64].

The terpenoid components in goji berries are significantly affected by cultivar, origin and processing methods. Authentic Ningxia cultivars contain higher levels of carotenoids, which is related to sufficient sunlight. Vacuum freeze drying is more conducive to component retention than high-temperature hot-air drying, and fermentation can improve the extraction efficiency of triterpenoids, providing theoretical support for process optimization and variety breeding [65].

### 5.8. Vitamins

Vitamins are indispensable organic substances for maintaining human life activities. Although their contents in the body are low, they play critical roles. Most vitamins cannot be synthesized by the human body and must be obtained from the diet. Fruits are important sources of vitamins, including vitamin C (ascorbic acid), vitamin A, B vitamins (thiamine, riboflavin, niacin, pyridoxine, etc.) and vitamin E. Most of these vitamins participate in the regulation of human metabolism in the form of coenzymes, which are essential for maintaining metabolic homeostasis.

Goji berries are rich in various vitamins, especially vitamin C, vitamin A (converted from carotenoids) and B vitamins (thiamine, riboflavin, niacin, pyridoxine). Among the vitamins in goji berries, vitamin C has been the most extensively studied. It exhibits excellent antioxidant properties, scavenges free radicals, enhances immunity, and inhibits the formation of nitrosamines in processed products, thereby improving product safety. According to research data, the recommended daily intake of vitamin C for adults (75–90 mg) can be basically met by consuming approximately 20–25 g of dried goji berries, highlighting the advantage of goji berries as a supplementary source of vitamin C.

Studies have shown that drying processing, regardless of pretreatment methods, leads to a significant decrease in vitamin C content in goji berry pulp, mainly because vitamin C is highly reductive and prone to oxidative degradation during drying. However, drying combined with ultrasonic pretreatment can effectively reduce the dissolved oxygen content in the pulp, thereby inhibiting the oxidative degradation of vitamin C and better retaining its content [66].

### 5.9. Bioelements and Other Elements

Similar to many fruits, natural goji berries are rich in various bioelements including potassium, calcium, magnesium, sodium, phosphorus and iron in the pulp, peel and seeds. In addition, important nutritional elements such as manganese, zinc and copper have also been detected in goji berries. These bioelements participate in diverse physiological processes in the human body, such as maintaining bone health, regulating electrolyte balance and modulating enzyme activities, constituting an important part of the nutritional value of goji berries [67].

Compared with conventional fruits such as persimmon, banana and lemon, goji berries stand out as a superior natural source of essential mineral elements. They exhibit particularly prominent advantages in the accumulation of potassium, zinc and magnesium, which are closely related to human physiological metabolism and nutritional value. Extensive scientific studies have consistently indicated that potassium is the most dominant macroelement in goji berries, with its content far exceeding other mineral components. Following potassium, phosphorus, calcium and magnesium are also present at relatively high levels. Importantly, these four major mineral elements together account for more than 80% of the total mineral composition, highlighting the excellent mineral resource characteristics and nutritional value of goji berries.

In addition, the mineral element profiles of goji berry leaves are significantly influenced by soil environmental conditions. Previous research has demonstrated that high aluminum levels in soil can markedly reduce calcium accumulation while significantly increasing aluminum uptake in goji leaves, which may adversely affect normal plant growth and development. Fertilization regimes also play an important regulatory role in determining leaf elemental composition. For instance, increasing nitrogen application rates significantly elevate leaf nitrogen content but tends to decrease phosphorus and calcium concentrations. These results provide a scientific basis for optimizing soil management and fertilization strategies to promote growth performance and enhance the nutritional quality of goji berries [68].

### 5.10. Critical Notes on Methodological Variability

Substantial methodological heterogeneity exists across published studies on goji berry phytochemicals, which limits direct cross-study quantitative comparison. Differences in raw material origin, cultivar, harvest time, pretreatment and drying procedures, extraction parameters, and purity evaluation methods all contribute to large deviations in reported component contents and bioactivity data. For instance, the yield of ultrasound-assisted extraction varies by 20–40% depending on power and duration settings, and the biological activity of polysaccharides is highly dependent on molecular weight distribution, which is rarely uniformly reported in the literature. Standardized extraction protocols and structural characterization indicators are urgently needed to improve data comparability and reproducibility in future research.

## 6. Biological Activities

### 6.1. Antioxidant and Antiaging Effects

Numerous studies have verified that various tissues of goji berries, including the fruit, leaves, and root bark, exhibit remarkable antioxidant activity, and the related in vitro experimental data are summarized in Table 3. In multiple in vitro antioxidant evaluation systems, extracts from different goji berry parts exert excellent free radical scavenging and antioxidant capacities through diverse mechanisms. These include strong scavenging effects on 2,2-diphenyl-1-picrylhydrazyl (DPPH) radicals, 2,2′-azino-bis(3-ethylbenzothiazoline-6-sulfonic acid) (ABTS) radicals, hydroxyl radicals (OH·), increasing ferric reducing antioxidant power (FRAP), oxygen radical absorbance capacity (ORAC), nitric oxide (NO) radicals, and hydroxyl radicals; significant hydroxyl radical scavenging capacity (PhOH-RSC, H_2_O_2_ photolysis) and hydroxyl radical prevention capacity (FeOH-RSC, Fenton reaction); as well as obvious iron chelating activity (ICA). Together, these results fully illustrate the broad and potent antioxidant potential of goji berry extracts [69].

The antioxidant activity of goji berries is significantly affected by processing, storage conditions and tissue differences. Obvious distinctions exist between fresh and dried goji berries. Specifically, fruits dried at low temperature and stored for 6 months show the optimal radical scavenging activity. Appropriate low-temperature and dark storage maintains the structural stability of LBPs and moderately enriches antioxidant components such as carotenoids and phenols, further enhancing biological activity. In addition, LBPs extracted by yeast fermentation (LBP-Y) display significantly stronger antioxidant activity than those obtained by traditional hot-water extraction (LBP-W), with superior scavenging capacity against DPPH and superoxide anion radicals [74]. Notably, the antioxidant activity of goji berry leaves is significantly higher than that of fruits regardless of fresh or dried state. The antioxidant activity of goji berries mainly relies on three core bioactive components: phenolic compounds, carotenoids and goji berry polysaccharides. The total phenolic content and total carotenoid content are significantly positively correlated with antioxidant efficacy. At the cellular level, characteristic phenolic components such as chlorogenic acid and caffeic acid act as hydrogen or electron donors to participate in redox reactions, neutralize oxidative intermediates produced at the peroxidase active sites of COX-1 and COX-2, and alleviate cellular oxidative damage [75]. Moreover, ethanol extracts of goji berries rich in phenols and carotenoids inhibit excessive nitric oxide (NO) production in LPS-induced macrophages, exerting synergistic protective effects by regulating the crosstalk between oxidative stress and inflammatory responses. In vivo experiments have verified their antioxidant and anti-inflammatory effects, although the underlying molecular signaling pathways remain to be further elucidated [76].

LBPs and their complexes represent another crucial material basis for antioxidant activity, which varies with polysaccharide structures. For instance, the LBP-C1 fraction isolated from goji berries in the Qaidam Basin shows the strongest antioxidant potential by regulating the Nrf2/HO-1 signaling pathway to alleviate H2O2-induced oxidative stress in HepG2 cells [77]. Polysaccharide–protein complexes and oligosaccharides from goji berries also exhibit favorable antioxidant activity. The DPPH radical scavenging rate of fruit oligosaccharides displays obvious dose dependence: 18.32% at 10 mg/mL and up to 76.89% at 50 mg/mL. Oligosaccharides from goji berry leaves present similar activity, suggesting that oligosaccharide components can serve as potential natural antioxidants [78].

These bioactive components (polysaccharides, carotenoids, flavonoids, etc.) exert potent antioxidant effects by scavenging excessive free radicals and inhibiting lipid peroxidation, thereby delaying organismal aging. Meanwhile, goji berries promote glycogen storage in muscle and liver, enhancing physical vitality and providing physiological support for antiaging effects. Genomic studies have verified that 58 out of 270 identified bioactive components in goji berries are associated with 90 human aging-related genes, clarifying the material basis for its antiaging function [74]. A research team from the Chinese Academy of Sciences has decoded the high-quality genome of goji berries and revealed the complete biosynthetic pathway of goji berry pectin polysaccharides (LBPPs) for the first time, identifying the key rhamnosyltransferase gene RRT3020 that promotes LBPP production, offering a molecular basis for the regulation and utilization of these active components [79].

Specific studies demonstrate that pigments from Lycium ruthenicum Murray extracted by ultrasound–microwave synergistic extraction effectively scavenge DPPH·, hydroxyl and superoxide anion radicals in a dose-dependent manner. Goji berry leaf flavonoid oral solution significantly increases the activities of superoxide dismutase (SOD), catalase (CAT) and reduced glutathione (GSH) while reducing malondialdehyde (MDA) content in serum, liver and brain tissues of aging mice, improving oxidative stress status. Fourteen phenolic compounds identified in purified goji berry phenolics also show strong antioxidant activity [80]. Furthermore, goji berry polysaccharides scavenge hydroxyl radicals in mouse serum, inhibit MDA production in liver tissue, and reduce systemic oxidative stress, with a significant positive correlation between antioxidant activity and polysaccharide content, providing a new direction for exploring dose–effect relationships. In mouse oxidative injury models, LBPs significantly elevate SOD, glutathione peroxidase (GSH-Px) and CAT activities and decrease MDA levels in serum, liver, kidney and brain tissues, alleviating oxidative damage [81]. Another aging mouse model study confirms that long-term administration of *Lycium barbarum* water extract (LBW) downregulates the expression of atrophy gene homolog 1 (Atrogin-1) and muscle ring finger protein 1 (MuRF-1) via the forkhead box class o (FOXO) signaling pathway, modulates inflammation and reduces proinflammatory cytokine production through nuclear factor kappa B (NF-κB) and chemokine signaling pathways, decreases aging-related biomarkers such as advanced glycation end products (AGEs) and senescence-associated β-galactosidase (SA-β-gal), improves skeletal muscle morphology, enhances motor function, and exerts comprehensive antiaging effects [82].

Notably, most current antioxidant data are derived from in vitro chemical assays and rodent models, and the tested concentrations generally exceed those achievable by routine dietary intake in humans. The in vivo antioxidant efficacy and dose–effect relationship in humans remain to be further verified.

### 6.2. Anti-Inflammatory Activity

**Figure 4:**
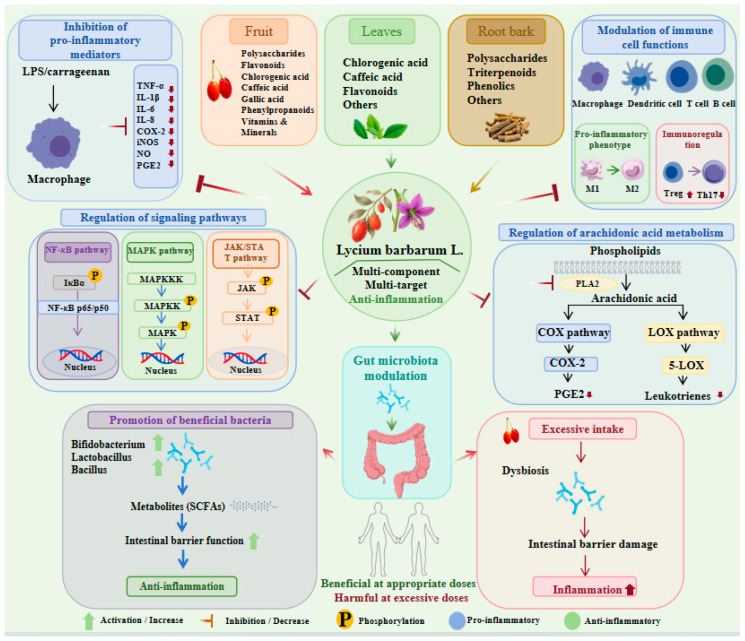
Regulation of anti-inflammatory signaling pathways in goji berries. This diagram is a conceptual summary of the regulatory pathways reported in the existing literature.

Epidemiological, toxicological and nutritional studies have confirmed that fruit consumption is closely associated with a reduced risk of chronic inflammatory-related diseases such as coronary heart disease, cancer, diabetes and neurodegenerative disorders. Phytochemicals, vitamins and minerals in fruits exert anti-inflammatory effects through multiple pathways, including inhibiting regulatory enzyme activities, scavenging free radicals, modulating arachidonic acid metabolism, regulating gene expression and immune cell functions. Goji berries and their extracts from different parts can exert significant anti-inflammatory effects by downregulating the levels of key proinflammatory cytokines such as interleukin 6 (IL-6), interleukin 8 (IL-8) and tumor necrosis factor-α (TNF-α), and the related regulatory mechanism is shown in Figure 4.

Studies have demonstrated that aqueous extracts of goji berries, leaves and root bark all exert ameliorative effects on inflammation-related diseases, and their anti-inflammatory activity is mainly attributed to active ingredients including flavonoids, phenolics (chlorogenic acid, caffeic acid, gallic acid), proanthocyanidins and goji berry polysaccharides. Moderate intake of goji berries helps maintain immune and inflammatory homeostasis in the body. Goji berry polysaccharides significantly inhibit the production of inflammatory mediators, including TNF-α, IL-6, nitric oxide (NO) and prostaglandin E2 (PGE2), and downregulate the gene expression of inducible nitric oxide synthase (iNOS) and cyclooxygenase-2 (COX-2) in lipopolysaccharide (LPS)-induced RAW 264.7 murine macrophage cell line (RAW 264.7) macrophages [83]. In addition, an appropriate dose of goji berry polysaccharides promotes the proliferation of beneficial bacteria such as Bifidobacterium, Lactobacillus and Bacillus in the intestinal tract, and short-chain fatty acids (SCFAs), their key microbial metabolites, play a critical role in regulating intestinal immunity for regulating intestinal immunity and exerting anti-inflammatory effects [84,85]. In contrast, excessive intake of goji berries may disrupt intestinal microecological balance, alter microbial community structure and metabolic characteristics, and thereby exacerbate colitis symptoms in mice.

Goji berry leaf extract is rich in phenolic components such as chlorogenic acid, caffeic acid and gallic acid and exerts anti-inflammatory and potential antitumor effects by regulating vascular endothelial growth factor (VEGF) and transforming growth factor-β (TGF-β) signaling pathways [29]. Meanwhile, goji berry root bark extract effectively inhibits λ-carrageenan-induced paw edema and reduces the excessive release of inflammatory factors including NO, interleukin 1β (IL-1β), IL-6 and COX-2 stimulated by LPS combined with λ-carrageenan, further reflecting its multi-target anti-inflammatory potential [76].

Current anti-inflammatory evidence is mainly based on cellular and animal models. The translation of these preclinical findings to human chronic inflammatory diseases requires further validation in well-designed clinical trials.

### 6.3. Blood Glucose Regulation

**Figure 5:**
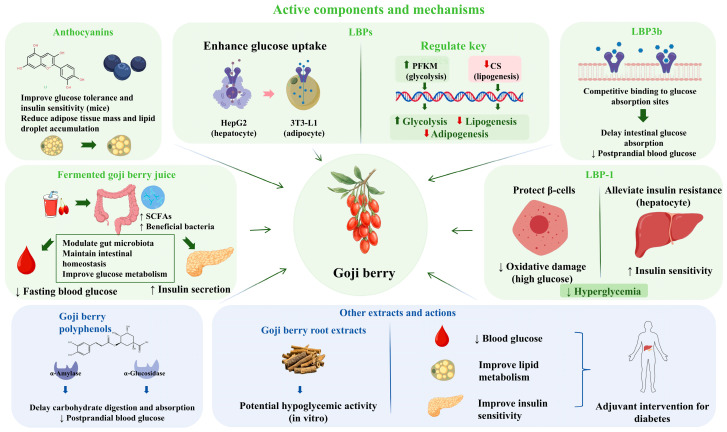
Mechanisms of Hypoglycemic and Metabolic Regulation by Bioactive Components of goji berries. This diagram is a conceptual summary of the regulatory pathways reported in the existing literature.

In recent years, the prevalence of diabetes has continued to rise with a trend toward younger onset. As a medicinal and edible homologous material, goji berries exhibit the advantages of high safety and mild effects. Their various active ingredients can improve glycemic control and enhance insulin sensitivity in diabetic patients through multiple targets and pathways, providing a natural and safe choice for adjuvant intervention of diabetes, and the related regulatory mechanism is shown in Figure 5.

Studies have confirmed that anthocyanins from goji berries significantly improve glucose tolerance and insulin sensitivity in C57BL/6 inbred mice, while reducing the volume and lipid droplet accumulation in inguinal white adipose tissue, providing animal experimental evidence for the regulatory effects of goji berries on glycolipid metabolism [86]. In in vitro cell experiments, treatment with LBPs markedly enhances glucose uptake in HepG2 human hepatocellular carcinoma cell line (HepG2) hepatocytes and 3T3-L1 murine preadipocyte cell line (3T3-L1) adipocytes [87]. Further mechanistic research reveals that LBPs upregulate the expression of PFKM, a gene encoding phosphofructokinase—a key enzyme in glycolysis—and simultaneously inhibit the expression of citrate synthase (CS), the gene encoding citrate synthase, which catalyzes the conversion of acetyl coenzyme A (acetyl-CoA) to citrate. These changes promote cellular glucose uptake, enhance glycolytic pathway activity, and suppress adipogenesis, thereby exerting synergistic effects in regulating blood glucose and blood lipids.

Notably, LBP fractions with different structures differ in their hypoglycemic mechanisms. In vitro cell experiments show that LBP3b, a polysaccharide fraction, inhibits cellular glucose uptake in a concentration-dependent manner. Its mechanism may involve competitive binding to glucose absorption sites, thereby delaying intestinal glucose absorption and reducing postprandial blood glucose levels [88].

In addition to active ingredients from goji berries, processing methods and extracts from different tissues also show significant hypoglycemic potential. Fermented goji berry juice significantly increases the content of SCFAs and the abundance of SCFA-producing beneficial bacteria, indirectly regulating blood glucose by modulating intestinal flora disorder and maintaining intestinal microecological homeostasis in diabetic mice. Experiments in diabetic rat models confirm that LBP intervention significantly inhibits pathological weight gain and reduces fasting blood glucose levels. More importantly, rats in the LBP-treated group exhibit significantly increased insulin secretion capacity under glucose stimulation, suggesting that LBPs can protect pancreatic β-cells from high-glucose toxicity and thereby improve systemic glycemic homeostasis [89].

Furthermore, in vitro hypoglycemic assays demonstrate that LBP-1, another polysaccharide fraction, reduces hyperglycemia by protecting pancreatic β-cells from oxidative damage and alleviating insulin resistance in hepatocytes. Goji berry phenolics of varying purities exert concentration-dependent inhibitory activities against α-amylase and α-glucosidase, assisting in lowering blood glucose by delaying carbohydrate digestion and absorption. Extracts from goji berry roots have also been verified to possess potential in vitro hypoglycemic activity, providing a new direction for the high-value utilization of the entire goji plant [90].

Most hypoglycemic studies are preclinical investigations. The effective doses in cells and animals are often higher than conventional dietary intake levels, so the clinical hypoglycemic efficacy in humans still needs large-sample trials to confirm.

### 6.4. Immunoregulation

**Figure 6:**
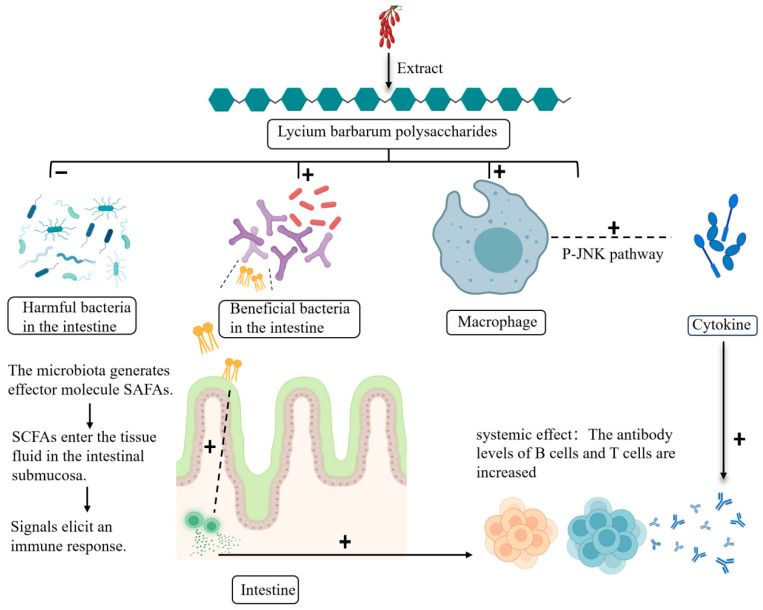
Regulation of immunomodulatory activity signaling pathways in goji berries.

The immune system serves as the core defense against invading pathogens, and its homeostasis relies on a sophisticated molecular and cellular network. Accumulating pharmacological evidence has demonstrated that numerous plant-derived bioactive components possess remarkable immunomodulatory potential. As a key immunoregulatory effector in Lycium plants, goji berry polysaccharides exhibit cultivar-specific structural features and biological activities, whose underlying mechanisms are illustrated in Figure 6. Specifically, goji berry polysaccharides exert upstream regulatory effects by reshaping intestinal microecological balance: they significantly inhibit the proliferation of intestinal pathogenic bacteria and promote the colonization of probiotics, thereby stimulating the production of short-chain fatty acids via microbial metabolism. As critical signaling molecules, these metabolites penetrate the intestinal mucosal barrier into the interstitial fluid, triggering local immune responses. At the cellular signal transduction level, goji berry polysaccharides act directly on macrophages by regulating the protein kinase B (Akt), NF-*κ*B and mitogen-activated protein kinase (MAPK) (ERK and p38) signaling pathway to optimize their phagocytic function and cytokine secretion profile, which further activates the downstream adaptive immune system in a cascade manner, promoting the proliferation and differentiation of B and T lymphocytes as well as antibody production, ultimately achieving systematic immune enhancement [91].

Processing techniques exert decisive effects on the structure–activity relationship of goji berry polysaccharides. Compared with hot-air drying, low-temperature freeze drying preserves the natural linear structure of polysaccharides to the greatest extent, thereby maintaining their basic immune activity; whereas the increased polymerization degree induced by hot-air drying reshapes their immunomodulatory function. Studies have shown that, although polysaccharides processed by different drying methods can both activate the canonical NF-*κ*B signaling pathway, they direct distinct immune response tendencies: freeze-dried samples tend to promote B-cell-mediated humoral immunity, while hot-air dried samples are more likely to induce T cell subset balance to strengthen cellular immunity. Furthermore, chemical modification significantly enhances polysaccharide activity. Sulfated goji berry polysaccharides exhibit superior immune-enhancing effects over natural polysaccharides by coactivating cluster of differentiation 40/8-6 (CD40/CD8-6) costimulatory molecules and inducing classically activated M1-type macrophage (M1-type) polarization of macrophages [92].

### 6.5. Hepatoprotective Activity

Goji berries exhibit significant hepatoprotective bioactivity in the prevention and treatment of liver diseases, which is mainly attributed to their multiple active components rich in goji berry polysaccharides, carotenoids (such as zeaxanthin and lutein), and phenolic acids (such as chlorogenic acid and caffeic acid). These components construct a three-dimensional defense network against liver injury through synergistic effects of multiple targets and pathways.

At the level of antioxidative stress, goji berry polysaccharides significantly upregulate the expression of key antioxidant enzymes in liver tissue, specifically enhancing the activities of SOD and glutathione peroxidase (GSH-Px), while effectively reducing the accumulation of lipid peroxidation products such as MDA. Such regulation of redox balance significantly alleviates oxidative damage to liver cell membranes and organelles caused by reactive oxygen species, thereby maintaining the structural and functional integrity of hepatocytes [93].

Against liver inflammatory responses, goji berries active components display distinct anti-inflammatory properties. Their mechanism involves inhibiting the activation of the NF-*κ*B signaling pathway, thereby downregulating the expression levels of proinflammatory factors including TNF-*α*, IL-1β and IL-6 in liver tissue. By reducing inflammatory cell infiltration, goji berry extract effectively relieves liver inflammatory injury and significantly improves inflammation-driven pathological changes such as alcoholic hepatitis and non-alcoholic fatty liver disease [94].

In terms of lipid metabolism regulation, goji berry polysaccharides promote hepatic fatty acid β-oxidation by activating the peroxisome proliferator-activated receptor α (PPARα) signaling axis. At the same time, they inhibit the expression of key lipid synthesis genes such as acetyl-CoA carboxylase and fatty acid synthase, thereby blocking ectopic deposition of triglycerides in hepatocytes and markedly alleviating hepatic steatosis in non-alcoholic fatty liver disease models [95].

For chemical liver injury (e.g., injury induced by carbon tetrachloride or acetaminophen), goji berry extract exerts protective effects mainly by interfering with the apoptotic cascade. Studies have shown that it regulates the Bcl-2/Bax protein ratio, inhibits the initiation of the mitochondrial apoptotic pathway, reduces hepatocyte apoptosis, and promotes the regeneration and repair of damaged liver tissue. This process is accompanied by a significant decrease in serum alanine transaminase (ALT) and aspartate transaminase (AST) levels, confirming its effective antagonism against liver damage caused by chemical toxins. Both in vitro and in vivo data indicate that the hepatoprotective effects of goji berries and their active components are dose-dependent, and appropriate intake holds important application potential for maintaining liver homeostasis and adjuvant therapy of liver diseases [96].

### 6.6. Neuroprotective Effects

Goji berries exhibit outstanding neuroprotective activity in the prevention and intervention of neurodegenerative diseases, and their pharmacological mechanism of synergistic regulation via multiple targets and pathways has been supported by extensive experimental evidence. Phenolic compounds (e.g., chlorogenic acid, caffeic acid), LBPs and carotenoids (zeaxanthin, lutein) are the key active components mediating neuroprotective effects, which maintain central nervous system homeostasis through multi-dimensional mechanisms including antioxidation, anti-inflammation, antiapoptosis, and promotion of neural repair.

In the regulation of oxidative stress, goji berry active components significantly enhance the activities of endogenous antioxidant enzymes such as SOD and GSH-Px in brain tissue and directly scavenge accumulated ROS in nerve tissue. By inhibiting lipid peroxidation and the production of MDA, goji berry extract effectively alleviates oxidative damage to neuronal cell membranes, mitochondria and DNA, thus preserving the structural integrity and normal physiological functions of neurons [97].

Against neuroinflammation, LBP displays remarkable anti-inflammatory properties in models of neurodegenerative diseases such as Alzheimer’s disease (AD) and Parkinson’s disease (PD). Its mechanism involves suppressing excessive activation of microglia and proliferation of astrocytes. By blocking the activation of inflammatory signaling pathways including NF-κB and MAPKs, LBP downregulates the expression of proinflammatory cytokines such as TNF-α, IL-1β and IL-6, thereby reducing the neurotoxicity of inflammatory factors and relieving chronic inflammatory injury in neural tissue [98].

In the regulation of cell apoptosis, goji berry components exert antiapoptotic effects by modulating the expression of Bcl-2 family proteins. Studies have shown that LBP upregulates the expression of the antiapoptotic gene Bcl-2, downregulates the proapoptotic gene Bax and the activity of caspase-3, inhibits the initiation of the mitochondrial-dependent apoptotic pathway, and reduces neuronal loss in key brain regions including the hippocampus and substantia nigra, thus improving learning and memory functions. In addition, LBP promotes the synthesis and secretion of neurotrophic factors such as brain-derived neurotrophic factor (BDNF) and nerve growth factor (NGF), activates the phosphatidylinositol 3-kinase/protein kinase B (PI3K/Akt) signaling pathway, facilitates neuronal growth, differentiation and axonal regeneration, and improves synaptic plasticity [99].

Both in vivo and in vitro experiments have verified the biological activity of goji berries’ neuroprotective effects. In animal models, goji berry extract intervention significantly improves the performance of dementia model mice in the Morris water maze test and enhances spatial learning and memory abilities. In cell experiments, goji berry active components effectively increase the survival rate of nerve cells and alleviate cell damage induced by neurotoxins such as β-amyloid (Aβ) and 1-methyl-4-phenylpyridinium (MPP+). These findings systematically reveal the multi-dimensional pharmacological mechanisms of goji berries in the prevention and treatment of neurodegenerative diseases, providing a solid scientific basis for their clinical application as a natural neuroprotective agent [100].

### 6.7. Antitumor Effects

**Figure 7:**
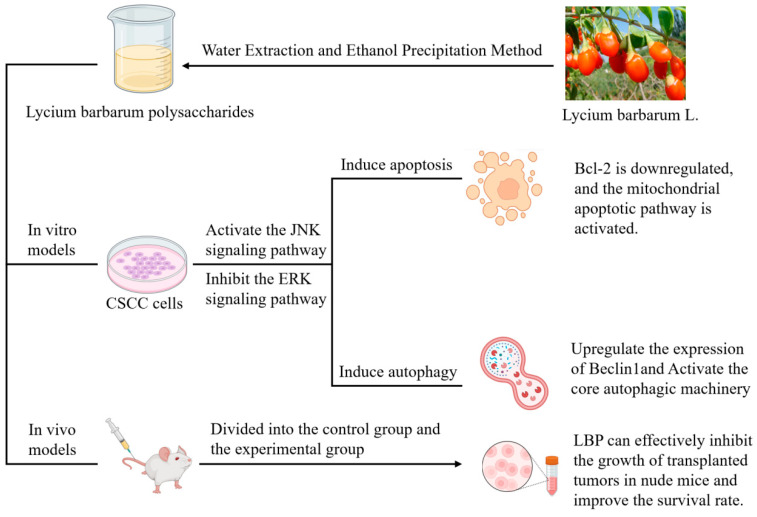
Antitumor mechanisms of LBPs. The pathways shown are based on reported preclinical mechanistic studies.

In recent years, malignant tumors have emerged as one of the major threats to public health. Conventional chemotherapeutic agents often exert significant toxic and side effects on the human body; therefore, exploring natural antitumor agents with high efficacy, low toxicity, and minimal adverse reactions has become increasingly crucial. As a traditional Chinese medicinal herb, goji berries have attracted extensive attention due to their potential antitumor properties. A large body of studies has demonstrated that various bioactive components contained in goji berries, such as LBPs and flavonoids, can exert antitumor effects through multiple mechanisms, including inhibiting tumor cell proliferation and metastasis, as well as inducing tumor cell death. LBPs exert antitumor activity by regulating multiple key signaling pathways, inducing tumor cell apoptosis and autophagy in vitro and inhibiting the growth of transplanted tumors in vivo. The specific action mechanisms are shown in Figure 7. Furthermore, the synergistic antitumor effects of bioactive components from goji berries combined with other therapeutic approaches have gradually become a research focus. Most findings remain at the preclinical stage and have not been fully validated in large-scale clinical trials.

#### 6.7.1. Direct Antitumor Mechanisms: Multi-Modal Cell Death Induction

LBPs are the key antitumor bioactive components in goji berries, which can induce tumor cell apoptosis, inhibit tumor cell proliferation, and exert antitumor effects by enhancing the body’s immune function. For instance, studies have shown that LBPs can inhibit the growth of solid tumors in mice [101]. Additionally, two LBP fractions, LBP-d and LBP-e, were isolated from goji berries, and their effects on human hepatocellular carcinoma SMMC-7721 cells were investigated separately. The results revealed that LBP-d could arrest SMMC-7721 cells at the G0/G1 phase with an inhibition rate of 26.70%, while LBP-e arrested the cells at the S phase with an inhibition rate of 45.13%. Moreover, the concentration of Ca^2+^ in the cytoplasm was increased in both cases, suggesting that LBPs may promote hepatocellular carcinoma cell apoptosis by regulating Ca^2+^ signaling pathways [102].

Relevant research on the antihepatocellular carcinoma activity of LBPs has confirmed that LBPs exhibit certain preclinical efficacy against hepatocellular carcinoma cells, as they can inhibit the proliferation and promote the apoptosis of cancer cells in a rat model of hepatocellular carcinoma. Transcriptome sequencing was used to analyze the differentially expressed genes in breast cancer cells before and after LBP treatment, and enrichment analysis was performed to explore the signaling pathways involved in the antitumor effect of LBPs. The results indicated that LBPs can inhibit breast cancer cells through the ferroptosis pathway; specifically, after treating MCF-7 and MDA-MB-231 cells with LBPs, the protein expression levels of xCT and GPX4 in the cells were significantly downregulated, suggesting that the mechanism may be associated with the xCT/GPX4 signaling pathway [103].

Other studies have found that LBPs can arrest tumor cells at the G0/G1 phase, thereby inhibiting tumor cell growth and exerting anticolorectal cancer effects [104]. It has also been reported that LBPs can inhibit the proliferation of gastric cancer cells by arresting them at the G0/G1 and S phases, exerting antitumor activity [105]. Research on human cutaneous squamous cell carcinoma has shown that LBPs can significantly inhibit the proliferation and induce the apoptosis of A431 cells, with a remarkable antitumor effect. These effects may be related to LBPs activating the MAPK/JNK pathway and promoting cancer cell autophagy, which is consistent with the core pathway regulatory mechanism of LBPs against cervical cancer [106].

In studies related to cervical cancer, the antitumor effect of LBPs is particularly clear. In vitro experiments using cervical squamous cell carcinoma (CSCC) cells as a model have confirmed that LBPs can exert antitumor effects by regulating the MAPK family signaling pathway, specifically by activating the JNK signaling pathway and inhibiting the ERK signaling pathway, thereby disrupting the balance between prosurvival and proapoptotic signals in tumor cells. Meanwhile, LBPs can downregulate the expression of the antiapoptotic protein Bcl-2, activate the mitochondrial apoptotic pathway to induce CSCC cell apoptosis, and upregulate the expression of the key autophagy protein Beclin1 to initiate the cellular autophagy mechanism, leading to autophagic death of CSCC cells and achieving dual killing of cervical cancer cells [106].

Furthermore, relevant studies have found that the pectin LRP3-S1 isolated from Lycium ruthenicum Murr. can significantly inhibit the proliferation of pancreatic ductal adenocarcinoma cells (AsPC-1, BxPC-3, and PANC-1) in a dose-dependent manner and specifically attenuate the invasion ability of BxPC-3 cells without exerting significant cytotoxicity on normal human pancreatic (HPDE6-C7) and liver (LO2) cells. In vitro, LRP3-S1 can downregulate the phosphorylation of key proteins in the FAK/AKT/GSK-3β and p38 MAPK signaling pathways, including p-FAK, p-AKT, p-GSK-3β, and p-p38 MAP kinase, thereby blocking tumor cell growth and invasion signals [107]. In a study on Hca-F tumor-bearing mice, it was found that LBPs could inhibit the growth of solid tumors in mice; compared with the control group, the spleen index and thymus index of mice in the LBP treatment group were significantly increased. When the LBP concentration reached a certain level, it could significantly promote the production of IL-2 and reduce the protein expression level of vascular endothelial growth factor (VEGF), indicating that its antitumor mechanism may be related to enhancing the cellular and molecular immune activity of tumor-bearing mice [108].

Phosphoproteomic analysis of non-small cell lung cancer cells after LBP treatment in vitro and in vivo revealed changes in the phosphorylation levels of major proteins related to apoptosis, suggesting that the molecular mechanism by which LBPs inhibit cancer cell proliferation may be through regulating the expression of apoptotic proteins in signaling pathways to induce cell apoptosis [109]. LBPs can inhibit the proliferation, migration, and invasion of human gastric cancer SGC-7901 cells in a concentration-dependent manner. In vitro, LBPs can downregulate the expression of MMP2 and MMP9 proteins, suppress the epithelial–mesenchymal transition (EMT) process by reducing Snail and Vimentin levels while increasing E-cadherin expression, and inhibit the phosphorylation of key proteins (PI3K and AKT) in the signaling pathway [110].

The role of goji berries in tumor radiotherapy has also been confirmed; experimental results showed that LBPs can enhance the antitumor effect of radiotherapy and significantly inhibit the decline in immunity caused by radiotherapy [111]. In addition, multiple studies have confirmed that LBPs can repair the damaged immune system, activate the body’s immune function, effectively eliminate residual tumor cells after surgery, radiotherapy, and chemotherapy, and prevent tumor recurrence. These findings suggest that future studies can focus on other types of tumors to explore the broad-spectrum antitumor activity of LBPs and investigate their common signaling pathways or mechanisms of action.

#### 6.7.2. Whole-Plant Antitumor Resources and Synergistic Combination Therapy

The combination of bioactive components from goji berries with conventional chemotherapeutic agents can produce favorable synergistic antitumor effects, which not only enhance the antitumor efficacy but also reduce the toxic and side effects of chemotherapeutic agents and, to a certain extent, decrease the multi-drug resistance of tumor cells to common chemotherapeutic agents. Relevant studies have found that oxaliplatin combined with LBPs promotes the apoptosis of HCT116-OXR cells, indicating that LBPs reverse colon cancer drug resistance by inhibiting the PMI/PI3K/AKT pathway, laying a foundation for exploring the molecular mechanism of LBPs in sensitizing chemotherapy.

The combination of LBPs with TRAIL inhibits the growth of MLL acute lymphoblastic leukemia cell lines KOCL44 and KOCL45, with significant cell apoptosis [112]. Combining LBPs with the chemotherapeutic agents temozolomide and bevacizumab resulted in a significant reduction in tumor weight in the group receiving LBPs combined with chemotherapy compared with the chemotherapy alone group. Studies have found that the combination of doxorubicin and LBPs with photothermal local therapy has significant advantages, improving the antitumor effect while reducing the major cardiac and systemic toxicity of doxorubicin and exerting a certain degree of anti-inflammatory and immune function.

LBPs also show great potential in the field of nanodrug carriers. For example, paclitaxel nanoparticles constructed with LBPs as carriers not only significantly enhance the antitumor efficacy of paclitaxel but also effectively reduce its toxicity during treatment, opening up a new path for the application of polysaccharide nanocarriers. A nanoparticle drug delivery system prepared by combining LBPs with photothermal materials makes full use of the antitumor activity of LBPs and photothermal therapy, thereby effectively improving the treatment effect of breast cancer [113].

Goji berries combined with existing antitumor therapies through multi-dimensional synergistic mechanisms show significant advantages in modern theoretical research and clinical trials, which is conducive to providing safer and more effective treatment options for tumor patients. Among them, the synergistic antitumor potential of LBPs in cervical cancer cells is worthy of further exploration. Combined with their core mechanisms of regulating the JNK/ERK pathway, inducing apoptosis and autophagy, LBPs can be combined with chemotherapeutic agents, photothermal therapy, etc. to further improve the treatment effect of cervical cancer and reduce toxic and side effects [114].

#### 6.7.3. Clinical Translation Status, Challenges and Future Directions

The clinical application of goji berries antitumor agents is currently limited by two major technical barriers: poor bioavailability and lack of tumor targeting. Nanomedicine provides an ideal solution to these challenges, and LBPs themselves have emerged as promising natural nanocarriers due to their excellent biocompatibility and inherent antitumor activity. LBPs can be self-assembled into nanoparticles (NPs) through electrostatic interaction or chemical crosslinking, which can encapsulate chemotherapeutic agents, photosensitizers, or nucleic acids. For example, paclitaxel-loaded LBP NPs showed 2.8-fold higher tumor accumulation than free paclitaxel in a breast cancer mouse model, with significantly reduced systemic toxicity. pH-sensitive LBP NPs further achieve controlled drug release in the acidic TME, improving therapeutic specificity.

Although most studies are still in the preclinical stage, preliminary clinical trials have shown encouraging results. A phase II clinical trial involving 120 patients with advanced non-small cell lung cancer found that adjuvant treatment with LBPs significantly improved patients’ quality of life, increased CD4+/CD8+ T cell ratios, and prolonged median progression-free survival from 3.2 months to 4.8 months compared with chemotherapy alone. Another study showed that LBP adjuvant therapy reduced the incidence of chemotherapy-induced leukopenia by 42% in gastric cancer patients. In terms of safety, goji berries have a history of more than 2000 years of edible and medicinal use, and clinical studies have confirmed that oral administration of LBPs at doses up to 3 g/day for 6 months is well-tolerated with no serious adverse reactions. The most common mild adverse reactions are gastrointestinal discomfort (1.2%) and dry mouth (0.8%).

Overall, the antitumor effects of goji berries described in this review are predominantly supported by preexperimental evidence, and their clinical therapeutic value requires more rigorous high-level clinical trials to confirm. Despite these promising advances, the clinical translation of goji berry antitumor agents still faces three key challenges: (1) lack of standardized quality control: the content and activity of bioactives vary significantly with origin, cultivar, and processing methods, and there is no unified quality standard specifically for antitumor products; (2) unclear molecular targets: the direct molecular targets of LBPs and other bioactives remain largely unknown, hindering the development of integrates processing–property–bioactivity associations; (3) insufficient high-quality clinical evidence: most clinical studies are small-sample, single-center trials with non-randomized designs, lacking large-scale phase III clinical trial data.

Future research should prioritize the following directions: (1) establishing standardized quality control systems for goji berry antitumor products, including specifications for LBP molecular weight distribution and monosaccharide composition; (2) identifying the direct molecular targets of LBPs using multi-omics technologies and CRISPR-Cas9 screening; (3) conducting multi-center, randomized, double-blind, placebo-controlled phase III clinical trials to validate efficacy and safety; (4) developing novel LBP-based nanodrug delivery systems for targeted tumor therapy.

### 6.8. Other Health Benefits

In addition to its significant bioactivities in immunoregulation, hepatoprotection and neuroprotection, goji berries also confer important health benefits via protecting the cardiovascular system and regulating intestinal health. These effects are mainly achieved through the multi-target regulation of metabolic pathways and microecological balance by their active components.

In terms of cardiovascular protection, goji berry polysaccharides serve as the core effector molecules, and their mechanisms involve systematic regulation of lipid metabolism. Studies have shown that goji berry polysaccharides significantly reduce serum levels of total cholesterol (TC), triglycerides (TG) and low-density lipoprotein cholesterol (LDL-C) and inhibit lipid deposition on the vascular wall, thereby reducing the formation of atherosclerotic plaques. Meanwhile, they increase the content of high-density lipoprotein cholesterol (HDL-C), enhance the body’s ability for reverse lipid transport and clearance, and delay the progression of atherosclerosis. Furthermore, phenolic compounds in goji berries (such as chlorogenic acid and caffeic acid) maintain the structural and functional integrity of vascular endothelium by suppressing oxidative stress and inflammatory responses in vascular endothelial cells. These components improve vasodilatory function, regulate blood pressure, and exert certain alleviating effects on hypertension and hypertension-related cardiovascular damage. Animal experiments further confirm that intervention with goji berry extract significantly reduces vascular stenosis in atherosclerotic animal models and lowers the risk of cardiovascular events.

In the regulation of intestinal health, goji berry polysaccharides play a key role, and their mechanisms are closely related to the precise modulation of intestinal microecology. As high-quality prebiotics, goji berry polysaccharides selectively promote the proliferation of beneficial bacteria such as Bifidobacterium and Lactobacillus, inhibit the growth of potential pathogenic bacteria including Escherichia coli and Salmonella, and optimize the intestinal flora structure. SCFAs, including acetate, propionate and butyrate produced by the fermentation of goji berry polysaccharides by beneficial bacteria, not only lower intestinal pH and improve the intestinal microenvironment but also strengthen the intestinal mucosal barrier function, reduce endotoxin translocation and leakage, and thus decrease the incidence of intestinal inflammation. In addition, goji berry polysaccharides directly promote the proliferation and repair of intestinal mucosal epithelial cells and enhance the expression of intercellular tight junction proteins, further improving the defensive efficiency of the intestinal physical barrier. Such positive regulation of the “gut–immune axis” endows goji berries with potential adjuvant therapeutic value in improving intestinal dysfunction, irritable bowel syndrome and other intestinal disorders.

## 7. Comprehensive Translational Prospects and Future Research Roadmap

As a paradigmatic medicine–food homologous plant with over 2000 years of ethnopharmacological history, goji berries have evolved from a traditional tonic food to a global research hotspot in natural products and functional foods. This review has systematically integrated global advances in its botanical characteristics, phytochemical profiling of the whole plant (fruit, leaves, flowers, stems, root bark), processing–property–bioactivity associations, and multi-target biological activities. Notably, we have clarified the direct and synergistic antitumor mechanisms of core bioactives centered on LBPs, filling the critical gap in existing literature that lacks a systematic framework for whole-plant utilization and translational research. This section summarizes the core findings of this review, proposes multi-dimensional industrial translational directions, dissects the common bottlenecks restricting clinical and industrial application, and presents an evidence-based future research roadmap to promote the sustainable development of the global goji berry industry.

### 7.1. Core Research Findings and Translational Value

This review provides three key academic contributions that form the foundation for translational applications:

Whole-plant phytochemical mapping: We systematically compared the distribution, content, and structural characteristics of core bioactive components (LBPs, phenolics, carotenoids, alkaloids) in all edible and non-edible parts of goji berries. We confirmed that non-edible by-products (leaves, stems, root bark) are rich in phenolics, flavonoids, and polysaccharides, with total phenolic content in leaves even exceeding that in fruits, demonstrating remarkable high-value utilization potential.

Processing–property–bioactivity associations elucidation: We clarified that drying methods (vacuum freeze drying vs. hot-air drying) and extraction techniques (ultrasound-assisted extraction vs. conventional hot-water extraction) significantly affect the molecular weight distribution, monosaccharide composition, and biological activity of LBPs. Vacuum freeze drying and ultrasound-assisted extraction were identified as optimal strategies to preserve the structural integrity and bioactivity of core components.

Multi-target biological activity system: We systematically deciphered the molecular mechanisms underlying the antioxidant, anti-inflammatory, hypoglycemic, immunomodulatory, hepatoprotective, neuroprotective, and antitumor effects of goji berries. In particular, we revealed that LBPs exert antitumor effects through four non-overlapping mechanisms (cell cycle arrest, apoptosis, ferroptosis, autophagy) and exhibit unique synergistic potential with conventional chemotherapeutic agents, providing a scientific basis for their application in chronic disease intervention and tumor adjuvant therapy.

These findings not only verify the scientific connotation of the traditional ethnopharmacological applications of goji berries but also provide a comprehensive theoretical reference and data support for the development of goji berries as functional foods, natural health products, and clinical adjuvant therapeutic drugs.

### 7.2. Multi-Dimensional Industrial Translational Directions

Based on the core findings of this review, goji berries have broad translational prospects in three major industrial fields, which can significantly increase their economic value and promote the sustainable development of arid and semi-arid regions.

#### 7.2.1. High-Value Circular Utilization of Whole-Plant Resources

Currently, the goji berry industry mainly focuses on fruit processing, while a large amount of leaves, stems, flowers, and root bark are discarded as waste, resulting in serious resource waste and environmental pollution. According to the phytochemical data presented in this review, different parts of L. *barbarum* can be developed into targeted high-value products:

Fruits: As the core raw material, they can be processed into high-end functional foods, dietary supplements, and natural pigments (zeaxanthin) for the nutrition and health market.

Leaves: Rich in phenolics and dietary fiber, they can be developed into natural antioxidants, weight management products, and functional teas, addressing the growing demand for clean-label food ingredients.

Root bark: Containing unique alkaloids (kukoamine A, lyciumamide) and triterpenoids, they can be used as raw materials for traditional Chinese medicine preparations and natural antihypertensive products.

Flowers and stems: Rich in flavonoids and polysaccharides, they can be used in the development of cosmetics and feed additives, extending the industrial chain.

This whole-plant utilization model conforms to the global trend of circular economy and sustainable agriculture and can increase the comprehensive income of goji berry growers by more than 30%.

#### 7.2.2. Standardized Processing Technology System Construction

The quality of goji berry products is highly dependent on processing methods, as demonstrated in this review. Currently, the lack of standardized processing technology leads to significant differences in the content and activity of bioactive components between different batches of products. To address this issue, it is urgent to establish a full-process standardized processing system covering:

Harvesting and pretreatment: Standardize the optimal harvesting time for different parts and varieties based on their dynamic changes in bioactive components during fruit development.

Drying technology: Promote vacuum freeze drying and low-temperature hot-air drying technologies to maximize the retention of LBPs, carotenoids, and phenolics.

Extraction and purification: Popularize green and efficient extraction technologies such as ultrasound-assisted enzymatic extraction to improve extraction yield and product purity while reducing organic solvent usage.

The establishment of this standardized system will ensure the stability and consistency of goji berry product quality, laying a foundation for their entry into the international high-end market.

#### 7.2.3. Precision Nutrition Product Development

Goji berries exhibit multiple health benefits through multi-target mechanisms, making them an ideal raw material for precision nutrition products targeting specific populations:

For tumor patients: Develop LBPs-based nutritional supplements to enhance immune function, alleviate chemotherapy-induced toxicity, and improve quality of life, as supported by the antitumor and immunomodulatory data presented in this review.

For diabetic patients: Develop functional foods rich in LBPs and phenolics to regulate blood glucose and lipid metabolism, based on their hypoglycemic mechanisms of promoting glucose uptake and protecting pancreatic β-cells.

For the elderly population: Develop antiaging and neuroprotective products to improve cognitive function and delay age-related degeneration, leveraging their antioxidant and neuroprotective effects.

For office workers: Develop eye-protection products rich in zeaxanthin and lutein to alleviate visual fatigue and protect retinal function.

These precision nutrition products will meet the diverse health needs of different consumer groups and significantly increase the added value of goji berry products.

### 7.3. Common Bottlenecks Restricting Clinical and Industrial Translation

Despite the promising research progress and industrial prospects, the clinical and industrial translation of goji berry bioactives still faces three common bottlenecks that are shared across all application fields:

#### 7.3.1. Lack of Unified and Exclusive Quality Control Standards

The content and activity of bioactive components in goji berries vary significantly due to origin, cultivar, planting conditions, harvesting time, and processing methods. For example, LBP content in high-quality Ningxia “Ningqi 1” fruits can reach 12% of dry weight, while that in common cultivars is only 5–8%. Currently, most commercial products only specify total polysaccharide content without considering molecular weight distribution, monosaccharide composition, or biological activity. There is no exclusive quality control standard for goji berry functional products, leading to uneven product quality and hindering consumer trust and market expansion.

#### 7.3.2. Unclear In Vivo Bioavailability and Metabolic Fate

Most bioactive components in goji berries, especially LBPs (10–1000 kDa), have poor oral bioavailability (generally less than 5%) due to their large molecular weight and complex structure. The in vivo metabolic fate of these components, including their degradation by gut microbiota, tissue distribution, excretion pathways, and the relationship between plasma concentration and biological efficacy, remains largely unclear. Individual differences in gut microbiota further complicate the prediction of in vivo efficacy, making it difficult to determine optimal clinical doses and administration regimens.

#### 7.3.3. Insufficient High-Quality Clinical Evidence

Most existing studies on goji berries are limited to in vitro cell experiments and in vivo animal models, with a lack of high-quality, large-sample, randomized, double-blind, placebo-controlled clinical trials. The effective dose, optimal administration frequency, and standardized treatment course for human use have not been clearly established. In addition, the long-term safety of high-dose use and potential interactions with conventional drugs have not been systematically and comprehensively evaluated, which directly limits the clinical application and regulatory approval of L. *barbarum*-derived products.

### 7.4. Evidence-Based Future Research Roadmap

To address the above bottlenecks, we propose a three-stage research roadmap based on the findings of this review, which balances scientific rigor with industrial feasibility:

#### 7.4.1. Short-Term Priorities (1–3 Years)

Establish a unified quality control system for goji berry bioactive components, including standard fingerprint maps, specifications for LBP molecular weight distribution and monosaccharide composition, and in vitro biological activity evaluation methods.

Optimize green and scalable extraction and purification processes to obtain high-purity, high-activity bioactive fractions with clear chemical composition.

Conduct systematic acute and subchronic toxicity studies to establish the safety window for human use of core bioactive components.

#### 7.4.2. Medium-Term Goals (3–5 Years)

Conduct multi-center, randomized, double-blind, placebo-controlled clinical trials to validate the efficacy of goji berry products in adjuvant therapy of tumors, diabetes, and neurodegenerative diseases and determine optimal clinical doses and treatment courses.

Elucidate the in vivo metabolic fate and molecular targets of core bioactive components using multi-omics technologies and stable isotope tracing methods.

Develop novel delivery systems (such as LBP-based nanoparticles) to improve the oral bioavailability and tumor targeting of bioactive components.

#### 7.4.3. Long-Term Vision (5–10 Years)

Develop first-in-class functional foods and natural health products based on goji berry bioactives, with clear health claims and clinical evidence.

Establish a circular economy model for the goji berry industry, achieving 100% utilization of all plant parts and zero waste discharge.

Integrate goji berries into the global precision nutrition and preventive healthcare system, making it a representative product of traditional Chinese medicine going global.

### 7.5. Concluding Remarks

Goji berries represent a perfect combination of traditional ethnopharmacological wisdom and modern scientific research. Their unique multi-target biological activities and excellent safety profile make them a valuable resource for the development of functional foods, natural health products, and clinical adjuvant therapies. While significant challenges remain in clinical and industrial translation, the rapid development of analytical chemistry, molecular biology, and nanomedicine will accelerate the transformation of preclinical discoveries into practical applications.

This review has provided a comprehensive and systematic framework for the research and development of goji berries, bridging the gap between basic research and industrial application. We hope that this work will stimulate further interdisciplinary research involving pharmacology, chemistry, agronomy, and clinical medicine, unlocking the full potential of goji berries and contributing to global public health and the sustainable development of arid regions.

## 8. Conclusions

Medicinal and edible plants represent an invaluable treasure trove for modern drug discovery and precision nutrition, yet their translational potential has long been hampered by fragmented research, lack of systematic property–bioactivity associations elucidation, and disconnection between basic science and industrial/clinical applications. As a paradigmatic representative of traditional Chinese medicine with over 2000 years of ethnopharmacological history, *Lycium barbarum* L. (goji berry) has gained global popularity, but a comprehensive framework linking its whole-plant phytochemical basis to biological functions, processing adaptability, and antitumor translational potential has remained conspicuously absent. This systematic review addresses this critical knowledge gap by synthesizing state-of-the-art advances across botany, phytochemistry, pharmacology, and translational medicine, establishing an integrated research paradigm spanning “whole-plant resource utilization; processing–property–bioactivity associations; multi-target biological mechanism; clinical translational application”.

Phytochemically, this work provides the most comprehensive mapping to date of core bioactive components (LBPs, phenolics, carotenoids, alkaloids) across all edible and non-edible tissues of goji berries (fruit, leaves, flowers, stems, root bark). We quantitatively demonstrate that processing methodologies—particularly drying techniques and extraction strategies—exert deterministic effects on the molecular weight distribution, monosaccharide composition, and spatial conformation of LBPs, the primary bioactive constituent. Notably, vacuum freeze drying and ultrasound-assisted enzymatic extraction are identified as optimal industrial processes that preserve both structural integrity and biological activity of core components. A groundbreaking finding of this review is the revelation that processing by-products (leaves, stems, flowers) possess comparable or even superior bioactive potential to fruits: leaves exhibit 2–3 times higher total phenolic content than fruits, while root bark contains unique alkaloids and triterpenoids with distinct pharmacological activities. This discovery challenges the traditional fruit-centric utilization model and provides a scientific foundation for developing a circular economy in the goji berry industry, with the potential to increase grower income by over 30% while eliminating agricultural waste.

At the pharmacological level, we systematically decipher the multi-target, multi-pathway regulatory network underlying the health benefits of goji berries. Beyond validating the scientific connotation of its traditional uses for nourishing the liver and kidney and improving eyesight, this review integrates scattered evidence into a coherent systems pharmacology framework: antioxidant effects are mediated by the synergistic action of phenolics, carotenoids, and LBPs via the Nrf2/HO-1 pathway; anti-inflammatory activity arises from downregulation of NF-*κ*B and MAPK signaling and modulation of gut microbiota; hypoglycemic effects involve enhanced glucose uptake, pancreatic β-cell protection, and regulation of key glycolipid metabolism genes. Most significantly, this work provides the first comprehensive and systematic analysis of the antitumor mechanisms of LBPs, delineating four non-overlapping direct killing pathways: (1) induction of mitochondrial-mediated apoptosis via Bcl-2/Bax modulation; (2) cell cycle arrest at G0/G1 or S phase; (3) triggering of ferroptosis through downregulation of the xCT/GPX4 axis; and (4) activation of autophagic cell death via the MAPK/JNK pathway. Furthermore, we highlight the unique synergistic potential of LBPs with conventional cancer therapies: they not only enhance the efficacy of chemotherapeutics and reverse multi-drug resistance but also alleviate myelosuppression and organ toxicity induced by radiotherapy and chemotherapy. These findings establish goji berries as a promising natural adjuvant for cancer treatment, offering a low-toxicity alternative to address the limitations of current oncological therapies.

Despite these remarkable advances, three fundamental bottlenecks remain that impede the clinical and industrial translation of goji berry bioactives. First, the lack of a standardized quality control system specific to functional products: current commercial standards only specify total polysaccharide content, ignoring critical structural parameters such as molecular weight distribution, monosaccharide composition, and protein conjugation that directly determine biological activity. This leads to enormous variability in product efficacy and erodes consumer trust. Second, the poor oral bioavailability and unclear in vivo metabolic fate of macromolecular components like LBPs: less than 5% of orally administered LBPs are absorbed intact, and their degradation by gut microbiota, tissue distribution, and direct molecular targets remain largely uncharacterized, making it impossible to establish precise dose–effect relationships. Third, the insufficiency of high-quality clinical evidence: most existing studies are small-sample, single-center, non-randomized trials, lacking large-scale phase III clinical trials with biomarker-based patient stratification, which is essential for regulatory approval and clinical adoption.

To overcome these challenges, we propose an evidence-based, three-stage research roadmap that balances scientific rigor with industrial feasibility. In the short term (1–3 years), priority should be given to establishing a unified quality control system incorporating structural fingerprints and in vitro biological activity assays, optimizing green and scalable extraction processes, and conducting comprehensive safety evaluations. In the medium term (3–5 years), efforts should focus on elucidating the in vivo metabolic fate and molecular targets of core bioactives using multi-omics and stable isotope tracing technologies, developing novel LBP-based nanodelivery systems to improve bioavailability and tumor targeting, and conducting multi-center randomized controlled trials to validate clinical efficacy. In the long term (5–10 years), we envision the development of first-in-class functional foods and natural pharmaceuticals based on goji berry bioactives, the establishment of a zero-waste circular economy model for the entire goji berry industry, and the integration of goji berries into global precision nutrition and preventive healthcare systems.

In conclusion, goji berries exemplify the perfect synergy between traditional ethnopharmacological wisdom and modern scientific research. This review bridges the long-standing gap between basic research and translational application, providing a comprehensive theoretical foundation and practical roadmap for the sustainable development of the global goji berry industry. The research paradigm established herein—integrating whole-plant resource utilization, processing–property–bioactivity associations elucidation, and multi-target mechanism dissection—serves as a valuable model for the modernization of other medicinal and edible plants. As we continue to unravel the molecular mechanisms underlying its health benefits and develop innovative delivery systems, goji berries are poised to make significant contributions to the prevention and treatment of chronic diseases, cancer adjuvant therapy, and the advancement of global public health.

## Figures and Tables

**Table 2:** Comparative Analysis of Extraction Techniques for LBPs.

Extraction Technique	Mechanism of Action	Advantages	Disadvantages	Applicability	Reference
Hot-Water Extraction (HWE)	Diffusion and osmosis of hot water into cells to dissolve polysaccharides.	1. Simplicity and low equipment cost. 2. Eco-friendly with no organic solvent residue. 3. Cost-effective for large-scale production.	1. Low efficiency and time-consuming (2–3 h). 2. High energy consumption. 3. High temperatures may cause thermal degradation of polysaccharide structure.	The fundamental method; often serves as a baseline or pretreatment step for combined techniques.	[37,38]
Enzymatic Extraction	Specific enzymes (e.g., cellulase, pectinase) degrade cell wall components to release polysaccharides.	1. Mild conditions preserve biological activity. 2. High specificity and extraction yield. 3. Environmentally benign (“Green Chemistry”).	1. High cost of enzyme preparations. 2. Strict requirements for pH and temperature. 3. High specificity limits universality; requires screening.	Ideal for extracting high-activity polysaccharides where structural integrity is paramount.	[37]
Ultrasonic-Assisted Extraction (UAE)	“Cavitation effect” generates mechanical shear forces to disrupt cell walls and accelerate mass transfer.	1. Rapid extraction (30–60 min) with high efficiency. 2. Reduced solvent consumption. 3. Higher purity (reduced protein content).	1. High shear forces may cause depolymerization (reduced molecular weight). 2. Coextraction of pigments may complicate decolorization.	Frequently combined with enzymatic methods to synergistically enhance yield and activity.	[38,39]
Microwave-Assisted Extraction (MAE)	Dielectric heating increases intracellular pressure, leading to cell rupture and content release.	1. Rapid and uniform heating (5–10 min). 2. Minimal solvent usage. 3. High extraction yield.	1. Risk of localized overheating causing degradation if power is uncontrolled. 2. Higher initial equipment investment.	Suitable for rapid processing; requires precise optimization of microwave power and duration.	[38]
Combined Extraction Methods (e.g., UAE–Enzyme)	Synergistic application of physical (ultrasound/microwave) and biological (enzymatic) techniques.	1. Synergistic effect maximizes extraction yield. 2. Balances efficiency with the preservation of bioactivity. 3. Significantly reduces processing time.	1. Complex optimization and operation. 2. Higher operational costs (equipment + reagents). 3.Technically demanding.	The optimal strategy for high-value product development and comprehensive laboratory research.	[39]

**Table 3:** Antioxidant activity of the goji plant.

Plant Part	Extraction Solvent	Assay	Result(s)	References
Fruit	Methanol/water (80/20, *v*/*v*), purified by solid phase extraction (SPE) with HLB cartridges, eluted with methanol	DPPH radical scavenging activity, ABTS radical scavenging activity, ORAC assay; OH radical scavenging capacity (PhOH-RSC, H_2_O_2_ photolysis), OH radical prevention capacity (FeOH-RSC, Fenton reaction)	DPPH: 13.9–18.5 μmol VCE g^−1^; ABTS: 54–61 μmol VCE g^−1^; ORAC: 180–260 μmol VCE g^−1^	[69]
Fruit	80% methanol, non-digested	DPPH radical scavenging activity, iron chelating activity (ICA); ferric reducing antioxidant power (FRAP), nitric oxide (NO) radical scavenging activity	DPPH EC_50_: 246 ± 8 μg/mL; ICA EC_50_: 105 ± 5 μg/mL; FRAP: 176.4 ± 1.1 μmol AAE/g extract; NO scavenging EC_50_: 998 ± 70 μg/mL (lower EC_50_ = stronger activity)	[70]
Fruit	Acetone/water/acetic acid (70:29.5:0.5), 3 h shaking + 12 h dark soaking	DPPH radical scavenging, ABTS radical scavenging, ferric reducing antioxidant power (FRAP)	DPPH: 16.07–17.47 μmol TE/g, average 16.65 μmol TE/g; ABTS: 53.92–64.38 μmol TE/g, average 59.14 μmol TE/g;FRAP: 2639.03–4651.04 mmol Fe^2+^ E/100 g	[46]
Leaves	50%, 70%, 100% ethanol; 100% ethanol extract with chlorophyll removal	DPPH radical scavenging activity, ABTS radical scavenging activity; ferric reducing antioxidant power (FRAP)	IC50 (mg/mL): DPPH: 50% ethanol 1.01 ± 0.07, 70% ethanol 0.82 ± 0.03, 100% ethanol 0.34 ± 0.02, dechlorophyllized extract 0.14 ± 0.05; ABTS: 50% ethanol 0.55 ± 0.01, 70% ethanol 0.49 ± 0.00, 100% ethanol 0.27 ± 0.00, dechlorophyllized extract 0.08 ± 0.05; Ascorbic acid (positive control): DPPH IC50 0.08 ± 0.00 mg/mL, ABTS IC50 0.02 ± 0.00 mg/mL; FRAP value (mmol Fe(II)/100g): 50% ethanol 0.15 ± 0.01, 70% ethanol 0.16 ± 0.00, 100% ethanol 0.37 ± 0.01, dechlorophyllized extract 1.28 ± 0.08	[71]
Leaves	95% methanol (Soxhlet extraction)	DPPH radical scavenging assay	DPPH free radical inhibition rate reached 69.43 ± 0.36% at the extract concentration of 1 mg/mL	[72]
Root bark	Ultrasonic-assisted hot-water extraction under optimal conditions	DPPH free radical scavenging assay; ABTS free radical scavenging assay; hydroxyl free radical scavenging assay	Reached 97% at 80 μg/mL, close to the scavenging effect of vitamin C (Vc) at 100 μg/mL; The scavenging rate reached 97% at 600 μg/mL, significantly higher than that of Vc (60% at 100 μg/mL); The scavenging rate reached 70% at 1 mg/mL, equivalent to the scavenging effect of Vc at the same concentration	[73]

FRAP—ferric reducing antioxidant power; ORAC—oxygen radical absorbance capacity; VCE—vitamin C; TE—Trolox equivalent; IC50—50% scavenging rate; EC50—50% scavenging rate; AAE—ascorbic acid equivalent; OH—hydroxyl.

## Data Availability

No data was used for the research described in the article.
